# Inhibition of Hsp110-STAT3 interaction in endothelial cells alleviates vascular remodeling in hypoxic pulmonary arterial Hypertension model

**DOI:** 10.1186/s12931-023-02600-5

**Published:** 2023-11-17

**Authors:** Congke Zhao, Xiangyang Le, Mengqi Li, Yuanbo Hu, Xiaohui Li, Zhuo Chen, Gaoyun Hu, Liqing Hu, Qianbin Li

**Affiliations:** 1 Department of Medicinal Chemistry, Xiangya School of Pharmaceutical Sciences, Central South University, Changsha, 410013 Hunan China; 2 Key Laboratory of Study and Discovery of Small Targeted Molecules of Hunan Province, Department of Pharmacy, School of Medicine, Hunan Normal University, Changsha, 410013 Hunan China; 3 Hunan Key Laboratory of Diagnostic and Therapeutic Drug Research for Chronic Diseases, Changsha, 410013 Hunan China; 4 Hunan Key Laboratory of Organ Fibrosis, Changsha, 410013 Hunan China; 5 Department of Pharmacology, Xiangya School of Pharmaceutical Sciences, Central South University, Changsha, 410013 Hunan China

**Keywords:** Pulmonary arterial Hypertension, Vascular remodeling, Heat shock protein 110, Protein-protein interaction, Pulmonary arterial endothelial cell

## Abstract

**Background:**

Pulmonary arterial hypertension (PAH) is a progressive and devastating disease characterized by pulmonary vascular remodeling which is associated with the malignant phenotypes of pulmonary vascular cells. Recently, the effects of heat shock protein 110 (Hsp110) in human arterial smooth muscle cells were reported. However, the underlying roles and mechanisms of Hsp110 in human pulmonary arterial endothelial cells (HPAECs) that was disordered firstly at the early stage of PAH remain unknown.

**Methods:**

In this research, the expression of Hsp110 in PAH human patients and rat models was investigated, and the Hsp110 localization was determined both in vivo and in vitro. The roles and mechanism of elevated Hsp110 in excessive cell proliferation and migration of HPAECs were assessed respectively exposed to hypoxia. Small molecule inhibitors targeting Hsp110-STAT3 interaction were screened via fluorescence polarization, anti-aggregation and western blot assays. Moreover, the effects of compound **6** on HPAECs abnormal phenotypes in vitro and pulmonary vascular remodeling of hypoxia-indued PAH rats in vivo by interrupting Hsp110-STAT3 interaction were evaluated.

**Results:**

Our studies demonstrated that Hsp110 expression was increased in the serum of patients with PAH, as well as in the lungs and pulmonary arteries of PAH rats, when compared to their respective healthy subjects. Moreover, Hsp110 levels were significantly elevated in HPAECs under hypoxia and mediated its aberrant phenotypes. Furthermore, boosted Hsp110-STAT3 interaction resulted in abnormal proliferation and migration via elevating p-STAT3 and c-Myc in HPAECs. Notably, we successfully identified compound **6** as potent Hsp110-STAT3 interaction inhibitor, which effectively inhibited HPAECs proliferation and migration, and significantly ameliorated right heart hypertrophy and vascular remodeling of rats with PAH.

**Conclusions:**

Our studies suggest that elevated Hsp110 plays a vital role in HPAECs and inhibition of the Hsp110-STAT3 interaction is a novel strategy for improving vascular remodeling. In addition, compound **6** could serve as a promising lead compound for developing first-in-class drugs against PAH.

**Supplementary Information:**

The online version contains supplementary material available at 10.1186/s12931-023-02600-5.

## Introduction

Pulmonary arterial hypertension (PAH) is a progressive and devastating disease, characterized by increased pulmonary arterial pressure (PAP) and pulmonary vascular resistance (PVR) [1]. Hemodynamically, PAH is defined as mean PAP (mPAP) > 20 mmHg with PVR > 2 Wood Units and pulmonary artery wedge pressure ≤ 15 mmHg at rest [2, 3]. It is widely recognized that the intrinsic pathological feature of PAH is vascular remodeling, involving deregulated proliferation and migration of human pulmonary arterial endothelial cells (HPAECs) and pulmonary arterial smooth muscle cells (HPASMCs) [4]. As the initial step in the pathogenesis of PAH, endothelial dysfunction plays a crucial role in mediating structural changes in the pulmonary vasculature [5]. Thus, increasing research attention has been attracted to the early stage of PAH with the hope of perfecting the prognosis of PAH treatment primarily by improving endothelial dysfunction [6–9]. With deeper understanding of PAH pathogenesis, some progresses were made in conventional therapies, which are mainly associated with targeting nitric oxide, endothelin and prostacyclin pathways [10–13]. Despite a dozen of drugs approved for PAH, a severe clinical condition still exists for the current therapeutic managements largely mediating their effect through pulmonary vasodilation [14, 15]. As such, new strategies from improving vascular remodeling at the early stage of PAH may open a potential window for PAH treatment.

Heat shock protein 110 (Hsp110, also known as Hsp105 or HSPH1) is a stress-inducible molecular chaperone that helps cells survive by preventing the aggregation of misfolded/unfolded proteins [16, 17]. Hsp110 expression was proved to promote stabilization of oncogenic proteins and has been linked with metastasis and poor prognosis in cancers [18–20]. Mechanistically, Hsp110 could enhance the Wnt/β-catenin pathway, directly bind to the signal transducer and activator of transcription 3 (STAT3) to enhance its phosphorylation, thus contributing to tumor growth [21, 22]. In addition, activation of STAT3 would result in acute lung injury, but inhibition of Hsp110 could alleviate the inflammatory processes by decreasing STAT3 phosphorylation [23]. Most recently, it was demonstrated that significantly increased expression of Hsp110 occurred in the pulmonary arteries of mice and HPASMCs under hypoxia, and knockdown of Hsp110 would alleviate hypoxia-induced PAH in mice [24], which unraveled the mysterious role of Hsp110 in inducing vascular remodeling.

At present, the principles of research targeting heat shock proteins (HSPs) is decreasing protein levels with small-molecule inhibitors or knocking down HSPs with small interfering RNAs [25]. However, there is restrictive marketing approval for these existing HSPs inhibitors (mainly Hsp90 inhibitors) due to limited clinical efficacy and off-target effects like potential toxicities and inevitable heat shock responses [25, 26]. Of note, disrupting the protein-protein interaction (PPI) between HSPs and their clients is emerging as a promising strategy for cancer therapy, which allows for targeting the non-conserved regions of the protein [27, 28]. Besides, inhibition of Hsp110-STAT3 interaction with the small molecule could effectively suppress cancer cell growth by decreasing phosphorylated STAT3 and c-Myc protein levels [29, 30].Nonetheless, it remains unclear whether Hsp110-STAT3 interaction plays an important role in pulmonary vascular remodeling.

In this work, we first demonstrated the significant upregulation of Hsp110 in the serum of PAH patient as compared with healthy individuals. It’s also observed that Hsp110 expression is markedly increased in pulmonary arteries and lung tissue of PAH rats, which was induced by hypoxia or monocrotaline (MCT). Moreover, it’s shown that human Hsp110 is mainly localized in HPAECs and is abnormally upregulated during the early phase of vascular remodeling. Our studies highlighted that activation of the Hsp110-STAT3 interaction would contribute to the development of pulmonary vascular remodeling, and compound **6** was successfully identified as lead compound against the Hsp110-STAT3 PPI. The in vitro and in vivo results further indicated that compound **6** displayed remarkable inhibitory activities against HPAECs proliferation and migration, and significantly decreased mean pulmonary artery pressure (mPAP) and vascular remodeling as well. Taken together, our work provides new insights into the role of Hsp110 overexpression in early-stage vascular remodeling, with potential to inspire targeting Hsp110-STAT3 PPI for the treatment of PAH.

## Materials and methods

### Chemicals and reagents

KNK437 (purity > 98%) was purchased from Energy Chemical and compounds **1–20** were synthesized before [14]. All the compounds were dissolved in dimethyl sulfoxide (DMSO). Paraformaldehyde, NaCl, KOH, KCl, Mg(OAc)_2_ and glycerol were purchased from Sigma-Aldrich (Santa Clara, CA, USA); Hepes, DTT and IPTG were purchased from Solarbio (Beijing, China); CCK-8 kit was purchased from Topscience (Shanghai, China); protein lysis buffer and chemiluminescent plus reagents were obtained from Beyotime Biotechnology (Shanghai, China); transfection reagent Lipo3000 were purchased from Thermo Fisher Scientific (Waltham, MA, USA); Green fluorescent protein (GFP)-tagged lentivirus (LV-HSPH1) and negative control lentivirus (CON522) were purchased from Genechem (Shanghai, China). All other reagents and chemicals were obtained from available commercial sources.

### Cell lines and culture

Human PAECs, PASMCs, HLF-1 and HCT116 cell lines were purchased from American Type Culture Collection (Manassas, VA, USA). HPAECs were cultured in DMEM/F-12 (1:1) supplemented with 10% FBS (Gibco, Grand Island, NY, USA) and 1% penicillin/streptomycin mixture (Gibco, Grand Island, NY, USA). HPASMCs were cultured in SMCM supplemented with 2% FBS (ScienCell, USA), 1% SMCGS (ScienCell, USA) and 1% penicillin/streptomycin mixture (ScienCell, USA). HLF-1 cells were cultured in DMEM/F-12 K (1:1) supplemented with 10% FBS (Gibco, Grand Island, NY, USA) and penicillin/streptomycin mixture (Gibco, Grand Island, NY, USA). HCT116 cells were cultured in DMEM supplemented with 10% FBS (Gibco, Grand Island, NY, USA) and penicillin/streptomycin mixture (Gibco, Grand Island, NY, USA).

### Elisa assay

The plasma samples from 10 patients, initially diagnosed with idiopathic PAH, (2 males and 8 females) aged 20–58 years old with a median of 44 years, healthy individuals (3 males and 7 females) aged 25–50 years old with a median of 40 years. All the patients received proper treatment including surgery or targeted drug therapy in Xiangya Hospital after plasma collection. This study was approved by the Medical Ethics Committee of Xiangya Hospital of Central South University and conducted in accordance with the Declaration of Helsinki. Written informed consent was obtained from each patient. The concentration of Hsp110 in human plasma was measured using a Hsp110 ELISA kit (Jingmei Biotechnology Co. Ltd, Jiangsu, China) according to the manufacturer’s instructions as previously described [31].

### Immunohistochemistry and histology staining

These staining measurements were performed according to previously report [31]. In short, rat lung were fixed with 4% paraformaldehyde and embedded in paraffin, which were cut into 5 μm sections. For immunohistochemisty staining, serial above sections were digested with 3% H_2_O_2_ for 20 min at room temperature, and then preincubated with 10% non-immunized serum. Sections were incubated with rabbit anti-Hsp110 antibody (1:100, Abcam, UK) overnight at 4 °C. The sections were incubated with biotinylated goat anti-rabbit secondary antibody (1:500, Santa Cruz) after washing the unbound antibodies, and then incubated with streptavidin-HRP. Subsequently, diaminobenzidine was used for color reaction to detect a positive signal according to routine procedure. Slides were photographed by a cell imaging multi-mode reader (Biotek Cytation5). Brown and yellow staining indicated positive results. For histology staining, the sections were stained with hematoxylin and eosin, and snapshots of histology were captured by the cell imaging multi-mode reader.

### Immunofluorescence

The method was performed as previously described [32]. In brief, the lung tissue sections were put onto gelatin-coated glass slides and permeabilized with 0.2% Triton X-100 for 20 min and blocked with 2% bovine serum albumin for 30 min. Then the tissues were incubated with Hsp110 antibody (1:100, Abcam, UK), α-SMA antibody (1:500, CST, USA) and CD31 antibody (1:800, CST, USA) overnight at 4 °C. Subsequently, the sections were washed three times with 1×PBS and then incubated with fluorescentlabeled secondary antibodies for 2 h. After washing with 1×PBS three times, the sections were stained nuclear with DAPI for 3 min at room temperature. The slides were covered with gelvatol and the immunofluorescence was visualized using a cell imaging multi-mode reader (Biotek Cytation5).

### Western blot analysis

The assays were conducted according to previously report [33]. Briefly, the concentrations of proteins extracted with RIPA buffer (Beyotime Biotechnology, China) including protease inhibitors and phosphatase inhibitors (Selleck, USA) were measured via the BCA Protein Assay Kit (KeyGEN, China). Protein samples were separated in SDS polyacrylamide gel and transferred to poly (vinylidene fluoride) membranes, which were blocked with 5% nonfat milk powder for 2 h at room temperature in Tris-buffered saline supplemented with 0.1% Tween 20. The membranes were incubated with primary antibodies overnight at 4 °C against the following proteins: anti-Hsp110 ( Abcam, UK); anti-Hsp70 (CST, USA); anti-Hsp90 (CST, USA); anti-STAT3 (CST, USA); anti-p-STAT3 (CST, USA); anti-c-Myc (CST, USA); anti-Cyclin D1(CST, USA); anti-PCNA (CST, USA); anti-β-actin (Proteintech, USA). Subsequently, the membranes were washed and incubated with secondary antibody for 2 h at room temperature. Finally, the bands were detected by a western fluorescent detection reagent (No. WBKLS0100, Millipore) and imaged within the ChemiDoc XRS + c imaging system (Bio-Rad).

### Knockdown assay

The knockdown assay was performed as previously described [31, 32]. The siRNA targeting Hsp110 was purchased from Ribobio (Guangzhou, China). HPAECs were seeded in 6-well plates and cultured to ~ 30% confluency. For the transfection, diluting the siRNA (20 µM) to a final concentration of 50 nM with transfected medium (Opti-MEM, Gibco, Grand Island, NY, USA) and Lipo3000 (Invtrogen, USA), which was gently mixed and incubated for 15 min at room temperature. Then cell culture medium was removed, the mixture and DMEM/F-12 (1:1) supplemented with 10% FBS (Gibco, Grand Island, NY, USA) were added into wells and incubated for 48 h. Sequence of siRNA targeting Hsp110 was as follows:

### 5’- GCTAGAAGCTTTCTATTCT-3’

#### Real-time quantitative PCR (RT-qPCR)

Total RNA was extracted from HPAECs using TRIZOL reagent (Invitrogen, Carlsbad, CA). Reverse transcription was performed according to the manufacturer’s protocol using the Takara RT kit (Takara, Dalian, China) with a reaction system containing 500 ng of total RNA in a volume of 10 µL. Real-time quantitative PCR (RT-qPCR) was performed using SYBR Premix EX TaqTM (TaKaRa). The expression level of Hsp110 was determined by RT-qPCR using GAPDH as a reference gene for the 2^−∆∆Ct^ method. Primers for RT-qPCR are listed in Table S1.

### Cell viability assay

For EdU assay, the *method* has been described before [34]. Briefly, HPAECs were plated into 96-well plates at a density appropriate about 4,000 cells/well. After cell adhesion, cells were treated with siRNA or compounds, and then cultured in incubators for 24 h at 37 °C. Then culture medium was replaced with fresh medium containing 10 µM EdU (Beyotime, China). After incubation for 3 h in incubators, the staining treatment was carried out on a cell imaging multi-mode reader (Biotek Cytation5). For CCK-8 assay, cells were seeded into 96-well plates at a density appropriate about 3,000 cells/well. After cell adhesion, HPAECs were treated with a range of concentrations of compounds for 48 h. Fresh CCK-8 (10 µL, 5 mg/mL, Biosharp) was added to each well and incubated at 37 °C for 2 h. The spectrophotometric absorbance of each well was measured at a wavelength of 450 nm. Three replicates were used for each treatment. The IC_50_ was calculated by GraphPad Prism 8 statistical software.

### Cell migration assay

The methods to test HPAECs migration via cell scratch and transwell chamber (with a pore diameter of 8 μm) experiments were reported previously [33]. For scratch assay, cells were cultured in 6-well plates, in which 2 × 10^6^ cells were seeded per well. After cell adhesion, straight scratches lines for each well were made using a sterile 200 µL pipette tip and washed with PBS three times. After 24 h under hypoxia (1% O_2_) treatment with siRNA or compounds in DMEM/F12 medium containing 1% FBS, pictures were captured from the same areas as those recorded at 0 h time points. Then the migration distances were analyzed by Image J software. For transwell assay, 1 × 10^4^ HPAECs treated with siRNA or compounds were seeded into the upper chamber containing the culture medium with 1% FBS per well, and 600 µL of complete medium was added to the lower chamber. After incubation for 24 h at 37 °C with 1% O_2_ and 5% CO_2_, the cells that migrated to the lower membrane were fixed with 4% paraformaldehyde and stained with crystal violet (Solarbio, China). Quantification was performed by observing and photographing the stained cells using a cell imaging multi-mode reader (Biotek Cytation5).

### Lentivirus construction and cell transfection

Green fluorescent protein (GFP)-tagged lentivirus (LV-Hsp110) and negative control lentivirus (CON522) were constructed and purified by Genechem (Shanghai, China). After 24 h, when the cell density was approximately 30%, the original medium was discarded and starved with serum-free medium for 12 h. Polybrene was diluted to 5 µg/mL with infection enhancer, then Hsp110 or blank lentivirus was added to make a lentiviral infection solution with MOI = 20 and mixed well. The plate was washed three times with PBS, then 1 mL of virus infection solution was added to each well, and after 12 h of infection, the plate was replaced with normal medium, and after 48 h, the transfection efficiency was observed by fluorescence. Finally, the samples were collected.

### Protein expression and purification

The human Hsp110 proteins were expressed using the pET-28a plasmid in the Novagen’s Rosetta2(DE3)pLysS strain (MilliporeSigma) and purified as previously described with some modifications [35, 36]. Briefly, Luria-Bertani (LB) medium containing kanamycin (50 µg/mL) was used to inoculate the fresh transformants untill the O.D. 600 reached around 0.6–0.8 at 37 °C after transformation. Then, induction was performed by adding IPTG to medium with the final concentration of 0.5 mM. Cells were harvested after induction for 12 h or overnight at 16 °C. After centrifugation, the pellet was resuspended in lysis buffer (25 mM Hepes-KOH, pH 7.5, 300 mM NaCl, 10% glycerol, and 1 mM DTT). The expressed His6-SUMO-Hsp110 fusion proteins were first purified on a HisTrap column. After eluting with a linear gradient of imidazole in lysis buffer,the fractions including pure His6-SUMO-Hsp110 fusion protein were pooled. The His6-SUMO tag was cleaved by Ulp1 protease and removed by a second HisTrap column. Then Hsp110 was further purified on a HiTrap Q column and eluted with gradient concentrations of NaCl. The peak fractions was concentrated to more than 10 mg/mL in buffer containing 25 mM Hepes-KOH, pH 7.5, 50 mM KCl, and 1 mM DTT. Finally, the purified Hsp110 protein was flash frozen in liquid nitrogen and stored in -80 °C freezer.

### Fluorescence polarization assay for determining ATP binding to Hsp110

The method was performed as previously described [35–37]. Briefly, a fluorescence polarization (FP) assay was performed to determine the binding affinity of Hsp110 for ATP using the fluorescence-labeled ATP (N6-(6-amino) hexyl-ATP-5-FAM, ATP-FAM, Jena Bioscience, Jena, Germany). For compounds screening, Hsp110 was diluted in the buffer (25 mM Hepes, 150 mM KCl, 10 mM Mg(OAc)_2_, 10% glycerol, and1 mM DTT) to 0.2 µM. Then, the mixture of Hsp110 proteins and each compound (final concentration: 100 µM) was incubated at the room temperature for 1 h. The ATP-FAM was added to the mixture at a final concentration of 20 nM, then the reactions were incubated for another 1 h at the room temperature, allowing ATP-FAM to bind to Hsp110 efficiently. The fluorescence polarization measurements were performed on a cell imaging multi-mode reader (Biotek Cytation5), and the reaction with 1% DMSO was used as a positive control. In order to test the IC_50_ of compound **6** against Hsp110, serial dilutions of **6** were incubated with Hsp110 for 1 h at the room temperature before ATP-FAM adding. Inhibition rates of each compound were measured using GraphPad Prism 8 software.

### Anti-aggregation activity of Hsp110

Hsp110 preventing firefly luciferase (Sigma-Aldrich, SRE0045) aggregation assay was carried out as previously described with minor modifications [35–38]. Before performing the assay, purified human Hsp110 was first treated with compounds at the room temperature for 2 h in buffer (25 mM Hepes-KOH, pH 7.5, 50 mM KCl, 5 mM Mg(OAc)_2_, 2 mM ATP, and 2 mM DTT). Then, the compounds-treated Hsp110 was mixed with luciferase in buffer, and the final concentrations of luciferase, Hsp110 and compounds were 2 µM, 1.5 µM and 100 µM, respectively. The mixture was plated 100 µL into per well in the 96-well plate, then cultured in incubators at 42 °C, and UV absorbance at 320 nm was monitored over time. Compounds were added in reaction buffer to achieve the indicated concentrations, with a held final concentration of 1% DMSO. Luciferase alone was used as a negative control, and luciferase with the addition of Hsp110 as a positive control. Anti-aggregation activities of Hsp110 and the UV absorbance at 320 nm were measured using GraphPad Prism 8 software.

### Surface plasmon resonance (SPR) analysis

This assay was performed as our previously report [39]. In brief, the binding of compound **6** or KNK437 to the Hsp110 protein was monitored with the OpenSPRTM system. According to the OpenSPRTM standard operating procedures, installing acid-coupling chip, running PBS (pH 7.4) at a maximum flow rate of 150 µL/min, loading 200 µL of 80% isopropanol (IPA) to remove bubbles, washing with buffer to evacuate air. Before reaching the baseline, binding studies were performed at a constant flow rate of 20 µL/min. Subsequently, 200 µL solution of EDC/NHS was loaded to the sample loop, then washed with buffer and evacuated with air. Injecting Diluted Hsp110 with buffer and run for 4 min, followed by injection of 200 µL of blocking solution. Then washing the sample loop with buffer to evacuate air. SPR binding data were obtained througth an appropriate gradient dilution series of compounds, and the injections were consecutively performed for 120–140 s association and 160 s dissociation, respectively. Finally, the affinity parameters were evaluated in a steady-state one-to-one analysis model using the TraceDrawer (Ridgeview Instruments ab, Sweden).

### Drug Affinity Responsive Target Stability (DARTS) analysis

Given the observation that some ligands can protect the target protein from degradation by proteases, DARTS assay has well been used to detect the interaction between a ligand and a protein when mixed in a solution [40, 41]. In short, about 2 × 10^7^ HPAECs were grown to 90% confluency in 10 cm cell culture dishes and lysed with M-PER lysis buffer (Thermo Fisher, USA) containing protease and phosphatase inhibitor (Selleck, USA) for 10 min at 4 °C. After centrifugation, the cell lysates were removed to new tubes and assigned to different groups, which were added with 10 × TNC buffer to afford 1 × TNC buffer (50 mM Tris-HCl, pH 8.0, 50 mM NaCl, 10 mM CaCl_2_). The mixture was treated with compound **6** (10 µM) and 1% DMSO respectively for 2 h at room temperature. Then each sample was treated with different concentrations of using pronase (Roche, Switzerland) for 30 min at 37 °C. The proteolysis was terminated by adding 5 × loading buffer and denaured for 10 min at 100 °C. Finally, the target protein Hsp110 was investigated by western blotting analysis.

### Co-immunoprecipitation (co-IP) assay

This assay was conducted as follows: Firstly, HPAECs and HCT116 cells were seeded into 10 cm cell culture dishes and incubated for 12 h to adherence at 37 °C with 5% CO_2_. Then, cells were treated with compound **6** (10 µM) and KNK437 (20 µM) for 48 h, respectively. Cells were lysed with IP buffer (Beyotime Biotechnology, China) at 4 °C for 15 min in the tube. The lysate was centrifuged at 4 °C in a rate of 12,000 rpm for 20 min, and the supernatants were taken into the corresbonding new tubes. The concentration of collected protein samples was measured through BCA method. The collected protein samples were incubated with mouse STAT3 antibody overnight at 4 °C before adding protein A agarose beads. After incubation 3 h at 4 °C, tubes were centrifuged at 4 °C in a rate of 8000 rpm for 2 min. The pellets were washed with PBS two times and denatured with loading buffer. Finally, the precipitated proteins were subjected to western blotting analysis with the indicated antibodies, including rabbit anti-Hsp110 (Abcam, UK); mouse anti-STAT3 (CST, USA); rabbit anti-β-actin (Proteintech, USA).

### Animal experiment

The specific scheme of animal experiment was carried out according to our previously reports [14, 33, 34]. Briefly, healthy male Sprague-Dawley (SD) rats (weighing 160–180 g) were purchased from Hunan SJA Laboratory Animal Co. Ltd. (NO: SYXK (Xiang) 2015-0017). All the experiments were approved by Hunan Normal University Experimental Animal Welfare Ethics Committee and Animal Management Committee (IRB approval number : D2021041). All rats were acclimated at 20–25 °C with 50-60% humidity for 7 days and then were randomly divided into 4 groups (*n* = 7 per group): (i) control group, rats were exposed to normobaric normoxia (21% O_2_) continuously for 4 weeks (ii) hypoxia group, rats were exposed to 10% O_2_ continuously for 4 weeks (iii) hypoxia plus KNK437 (15 mg/kg) treatment (3 weeks) group, and (iv) hypoxia plus compound **6** (15 mg/kg) treatment (3 weeks) group.

After PAH modeling, rats were anesthetized with 1% sodium pentobarbital (50 mg/kg, i.p.). Then, the mPAP was measured using a BL-420 F biological signal acquisition and analysis system (TECHMAN, Chengdu, China). A single lumen PE50 tubing (0.5 mm inner diameter, 0.9 mm outer diameter, SP0109, AD Instruments, Australia) filled with heparinized saline was inserted into the right external jugular vein, and the other end was connected to the measurement system. The mPAP trace curve was used to confirm the catheter position and representative shapes of the pressure was electronically measured. After that, the right ventricle (RV) and left ventricle (LV) plus interventricular septum (S) were dissected from the heart, which were weighed to evaluate RVH index through calculating the ratio of RV/(LV + S). Following hemodynamic measurements, lung tissues were fixed with paraffin and then stained with HE to measure morphology. The slices were examined under a cell imaging multi-mode reader (Biotek Cytation5) to visualize the morphology of pulmonary arterioles (diameter between 50 and 150 μm). The relative PAMT (%, distance between outer and inner elastic lamina) was calculated by 100 × PAMT/external diameter.

### Statistical analysis

All statistical tests were performed using GraphPad Prism 8.0 software (San Diego, CA, USA), and representative data were selected to create the figures. Statistical significance was determined either by the two-tailed Student’s t test (for two groups) or a one-way analysis of variance (ANOVA) (for three or more groups). A significant difference was assumed at the *P* < 0.05 and error bars represent SD of three experiments unless stated otherwise.

## Results

### Increased expression of Hsp110 in PAH

**Fig. 1:**
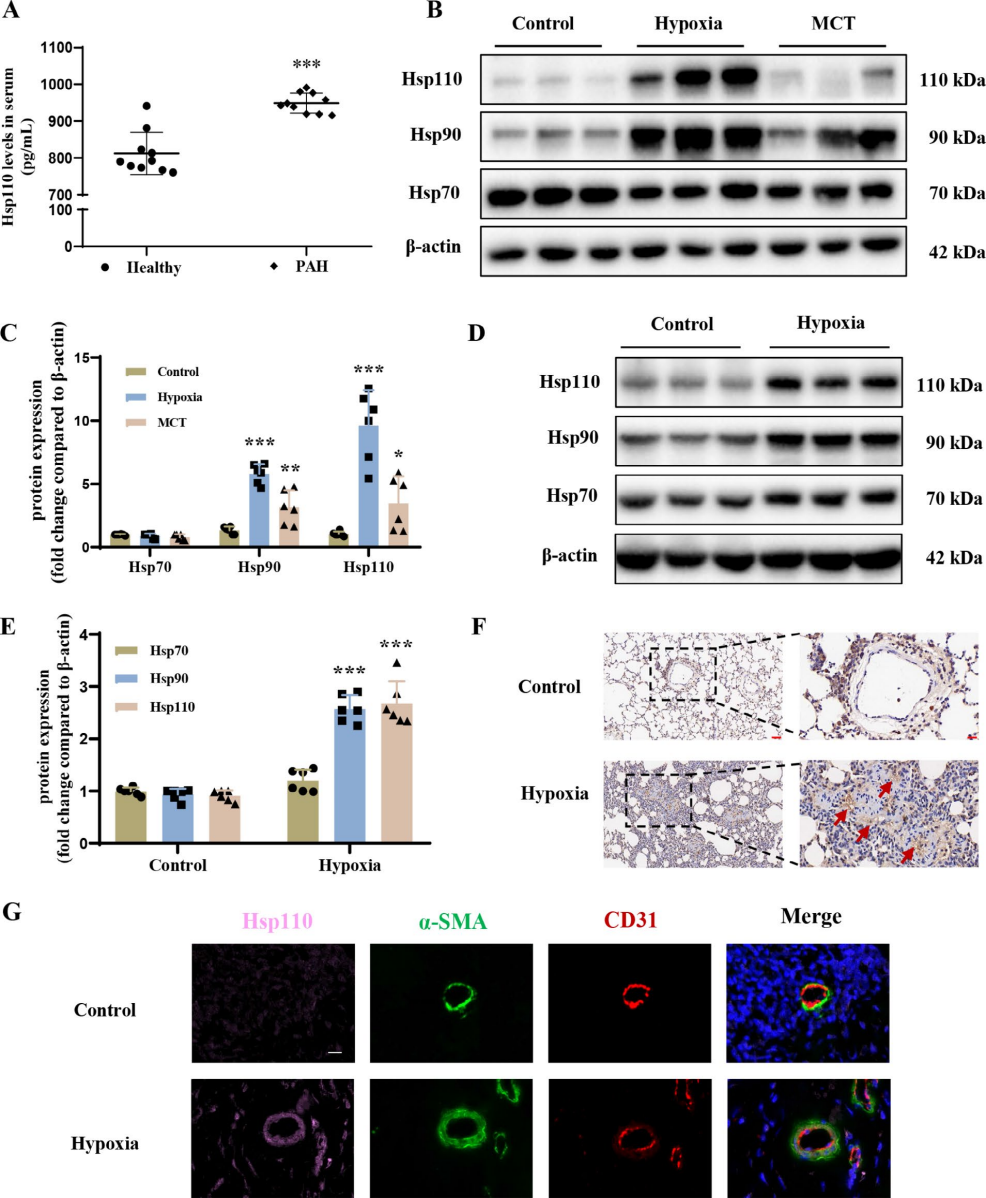
High expression and localization of Hsp110 in PAH. (**A**) Serum levels of Hsp110 in healthy subjects (*n* = 10), and PAH patients (*n* = 10). (**B**) Western blots of HSPs including Hsp110, Hsp90 and Hsp70 in lung tissues from PAH rat models (*n* = 6 each). (**C**) Statistical analysis of HSPs levels in lung tissues. (**D**) Western blots of HSPs in pulmonary arteries from hypoxia-induced PAH rat model (*n* = 6 each). (**E**) Statistical analysis of HSPs levels in pulmonary arteries. (**F**) Representative immunostaining micrographs of lung sections in hypoxia-induced PAH rats. Staining was performed for Hsp110. Scale bar: 50 μm (left), Scale bar: 20 μm (right). (**G**) Representative immunofluorescence staining of lung tissues for Hsp110 (pink), α-SMA (α-smooth muscle actin; green) and CD31 (red) from normoxia or hypoxia treated rats. Nuclei were counterstained with DAPI (blue). Scale bars: 25 μm. Results are expressed as the mean ± standard error; *P < 0.05, **P < 0.01, ***P < 0.001 *versus* the control group

To detect the expression of Hsp110 in PAH, we collected serum samples from patients with PAH and found by ELISA that serum Hsp110 levels were significantly upregulated in PAH patients compared to healthy individuals (Fig. 1A). To gain a detailed understanding of Hsp110’s role in PAH tissue, we investigated pulmonary tissues obtained from rats with PAH model induced by either hypoxia or MCT. Western blotting results showed that the upregulation of Hsp110 in the lung tissue was more pronounced in hypoxia-induced PAH rats compared to the MCT-treated group (Fig. 1B-C). Meanwhile, measurement of other heat shock protein subtypes was also conducted, such as Hsp90 and Hsp70. However, among these subtypes, Hsp110 expression exhibited the most significant upregulation. Moreover, western blot analysis of pulmonary arteries from hypoxic rats also confirmed a marked increase of Hsp110 when compared to the normal group (Fig. 1D-E). The immunostaining of pulmonary tissue sections revealed specific Hsp110 localization in the intimal layer of pulmonary vessels and further confirmed the elevated Hsp110 expression (Fig. 1F). In the concluding immunofluorescence co-localization study, Hsp110 was predominantly found in CD31-positive ECs with minimal presence in α-SMA positive SMCs (Fig. 1G). These findings imply a strong relationship between the upregulated expression of Hsp110 in pulmonary endothelial cells and the advancement of vascular remodeling in the context of hypoxia condition.

### Hypoxia-induced Hsp110 up-regulation affects the abnormal phenotypes of HPAECs

**Fig. 2:**
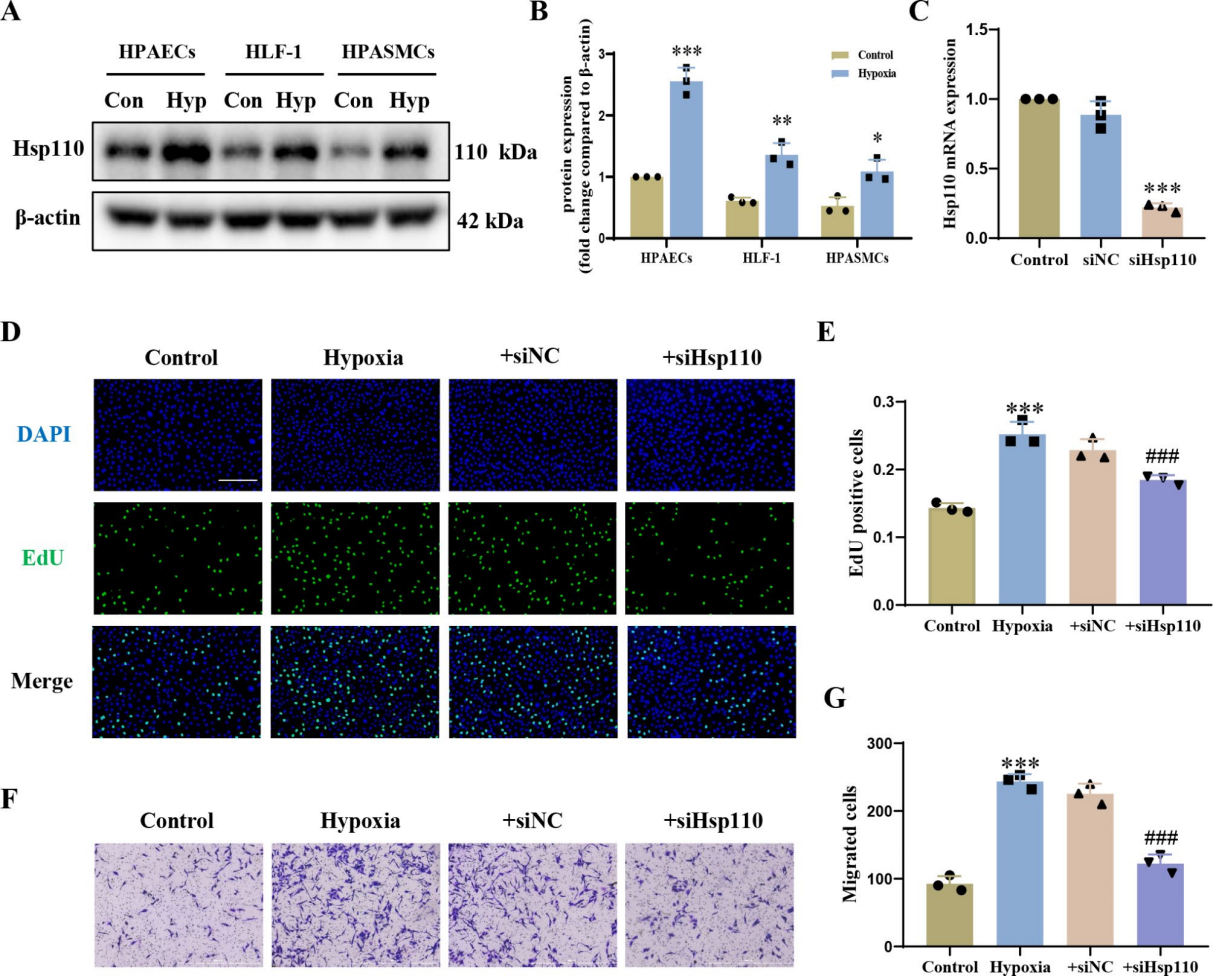
Expression of Hsp110 in HPAECs under hypoxia and its effect on hypoxia-induced proliferation and migration. (**A**) Western blots of Hsp110 in pulmonary artery cell lines under hypoxia, including HPAECs, HLF-1 and HPASMCs. Con: control, Hyp: Hypoxia (*n* = 3 each). (**B**) Statistical analysis of Hsp110 expression in pulmonary artery cell lines. (**C**) The knockdown efficiency of siRNA against Hsp110 in PAECs. (**D**) Images of proliferating nuclei labeled with 5-ethynyl-2’-deoxyuridine (EdU) in HPAECs under hypoxia with knockdown of Hsp110. Scale bars: 200 μm. (**E**) Statistical analysis of EdU assay. (**F**) Representative migration images from the trans-well assay in HPAECs under hypoxia with knockdown of Hsp110. Scale bars: 1000 μm. (**G**) Statistical analysis of trans-well assay. Results are expressed as the mean ± standard error, *n* = 3; *P < 0.05, **P < 0.01, ***P < 0.001 *versus* the control group, ^###^*P* < 0.001 *versus* the hypoxia group

To further study the role of Hsp110 in the behaviour of different cell types during vascular remodeling, we tested Hsp110 expression under hypoxia condition in three human pulmonary artery cell lines, including HPAECs, HPASMCs and HLF-1. The results demonstrated that Hsp110 was most significantly upregulated in HPAECs after hypoxia induction when compared with HPASMCs and HLF-1 (Fig. 2A-B). To examine the functional role of Hsp110 in the early stage of vascular remodeling, we applied siRNA to silence the expression of Hsp110 with a silencing efficiency of 78% (Fig. 2C). Afterwards, we performed EdU and Trans-well assay in HPAECs. Notably, the use of Hsp110 siRNA to suppress Hsp110 resulted in substantial inhibition of hypoxia-induced HPAECs proliferation and migration compared non-targeting siRNA (siNC) treated HPAECs, respectively (Fig. 2E-G). Collectively, these findings highlighted the influence of Hsp110 overexpression induced by hypoxia on the aberrant phenotypes of HPAECs, thus, Hsp110 inhibition might hold a promising way to mitigate endothelial dysfunction.

#### Elevated Hsp110 activates downstream STAT3 signaling pathway in HPAECs

**Fig. 3:**
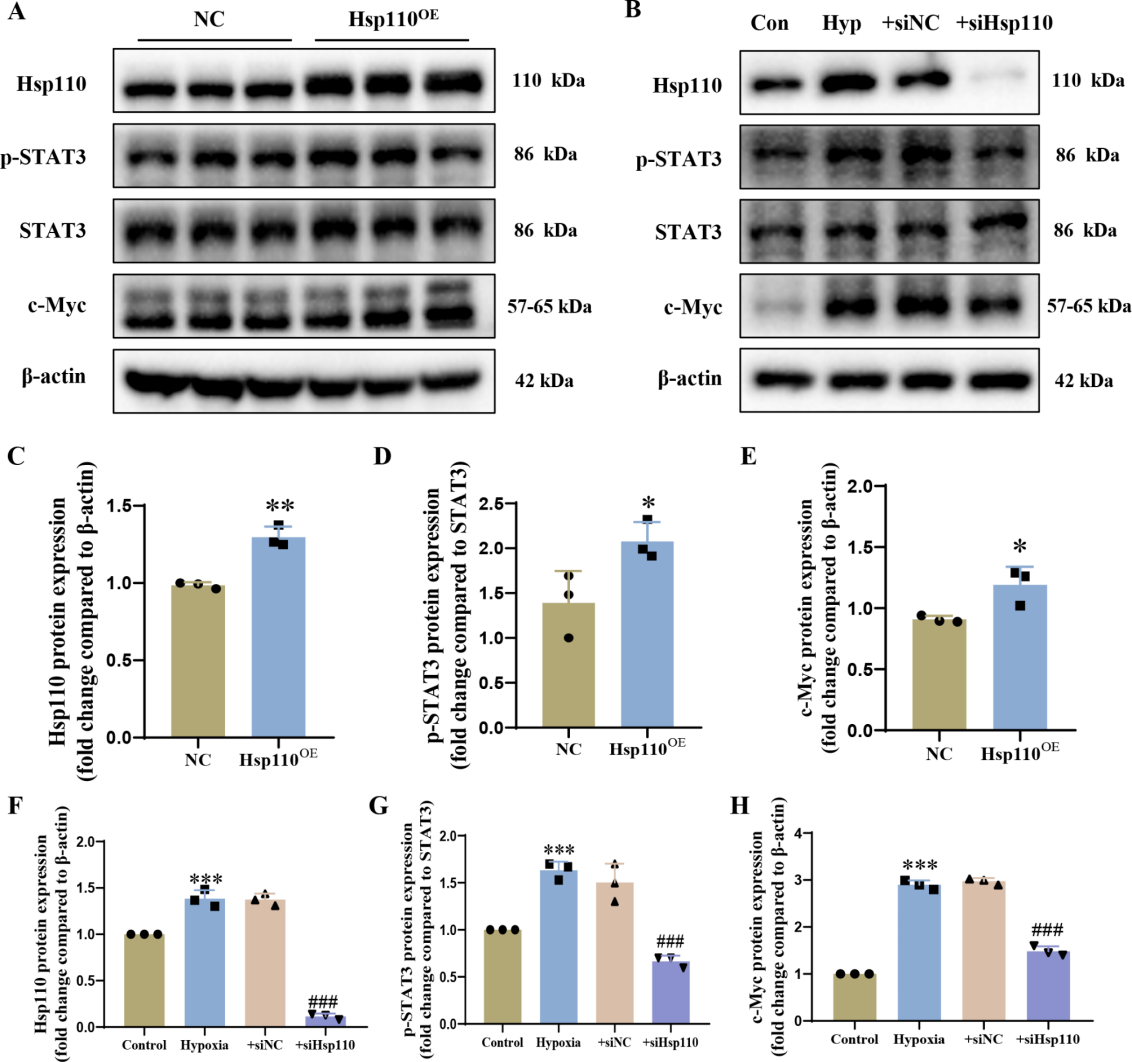
The regulatory mechanism of Hsp110 overexpression and knockdown on downstream STAT3 signaling pathway. (**A**) Western blots of Hsp110, p-STAT3, STAT3 and c-Myc in HPAECs after Hsp110 overexpression. (**B**) Western blots of Hsp110, p-STAT3, STAT3 and c-Myc in HPAECs under hypoxia with knockdown of Hsp110. (**C**) Statistical analysis of Hsp110 overexpression. (**D**) Statistical analysis of p-STAT3/STAT3 ratio after Hsp110 overexpression. (**E**) Statistical analysis of c-Myc levels after Hsp110 overexpression. (**F**) Statistical analysis of Hsp110 expression under hypoxia with knockdown of Hsp110. (**G**) Statistical analysis of p-STAT3/STAT3 ratio under hypoxia with knockdown of Hsp110. (**H**) Statistical analysis of c-Myc levels under hypoxia with knockdown of Hsp110. Results are expressed as the mean ± standard error, *n* = 3; *P < 0.05, **P < 0.01, ***P < 0.001 *versus* negative control (NC) or the control group, ^###^*P* < 0.001 *versus* the hypoxia group

Currently, Hsp110 up-regulation has well been reported to mediate the abnormal proliferation of cancer cells [22, 29]. In addition, studies on the mechanism of Hsp110 in tumor have shown that the combination of abundant Hsp110 with STAT3 causes activation of downstream STAT3 signaling via a specific increase in STAT3 phosphorylation [29]. Given the shared features between tumor cells and pulmonary endothelial cells in PAH patients, and the beneficial effects of the inhibitor treatment of Hsp110-STAT3 interaction in these diseases [27, 42], here we next tested the influence of Hsp110 on the regulation of the downstream STAT3 pathway in HPAECs. As shown in Fig. 3A, C-E, overexpression of Hsp110 by lentivirus resulted in an observed increase in p-STAT3 and c-Myc protein levels compared to the negative control. Additionally, western blotting analysis demonstrated a significant ascending of Hsp110 in HPAECs under hypoxia stimulation (Fig. 3B, F), and transfection of HPAECs with the Hsp110 siRNA contributed to a marked decrease in p-STAT3 and c-Myc levels (Fig. 3B, G-H). In summary, these studies suggest that upregulated Hsp110 induced by hypoxia activates the downstream STAT3 signaling pathway in HPAECs.

### Pyrazolo[3,4-*b*] pyridine derivative 6 was screened out as the inhibitor of Hsp110-STAT3 interaction

**Fig. 4:**
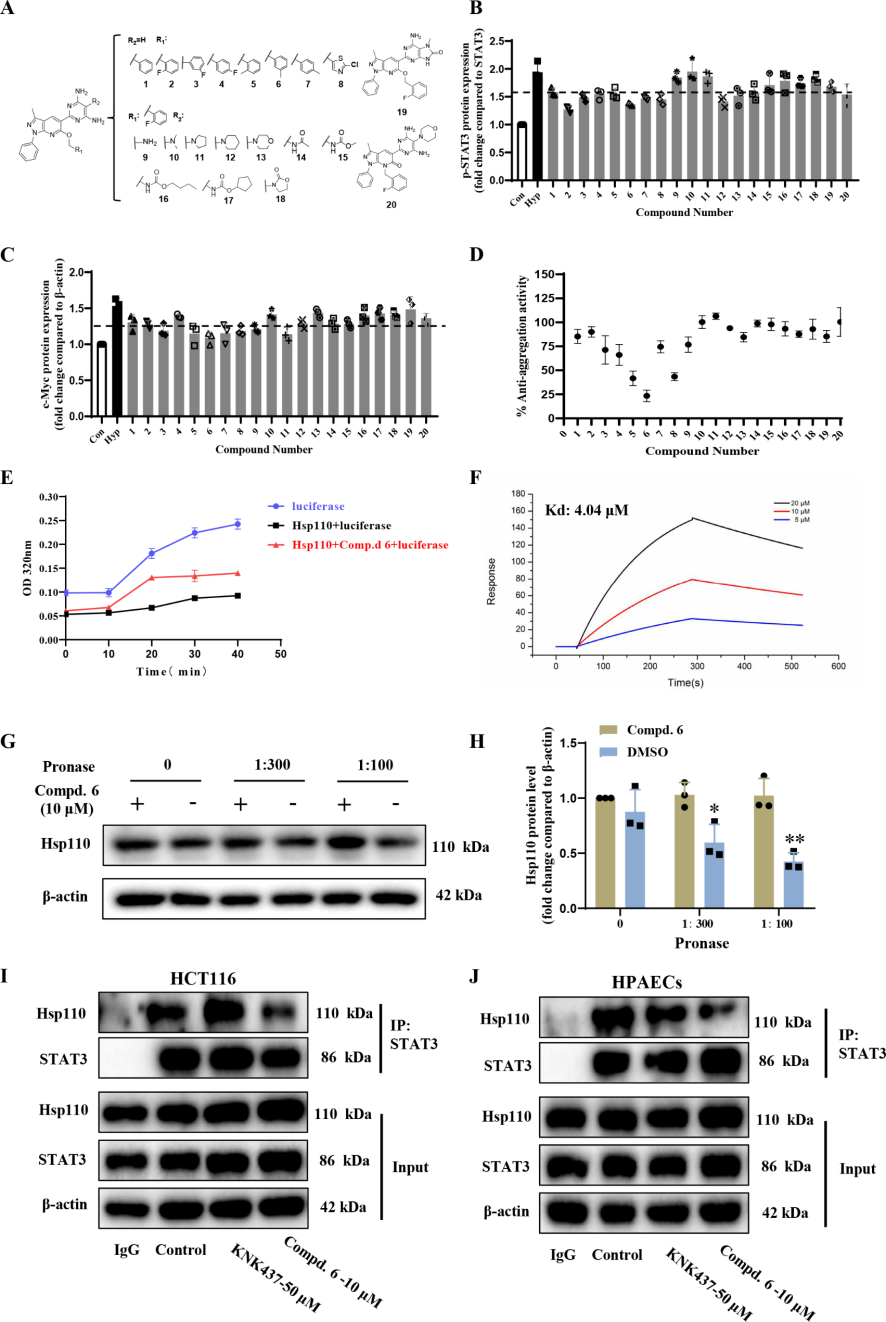
Compound screening targeting Hsp110 molecular chaperone function and Hsp110-STAT3 PPI. (**A**) The chemical structures of pyrazolo[3,4-*b*] pyridine derivatives evaluated in this study. (**B**) Compound screening results targeting the ratio of p-STAT3/STAT3 at 10 µM. (**C**) Compound screening results targeting the expression of c-Myc at 10 µM. (**D**) Compound screening results for Hsp110 anti-aggregation activity at 100 µM. (**E**) The time dependent effect of compound **6** on Hsp110 anti-aggregation activity at 100 µM. (**F**) SPR assay demonstrating the stable fit of the interaction between compound **6** and Hsp110 (Kd = 4.04 µM). (**G**) Compound **6** treatment increases the protease susceptibility of the Hsp110 in cell lysates as determined by the DARTS assay. (H) Statistical analysis of Hsp110 levels in DARTS assay. (**I**-**J**) Co-IP results indicating compound **6** inhibits the Hsp110-STAT3 PPI both in HCT116 cells and HPAECs. Results are expressed as the mean ± standard error, *n* = 3; *P < 0.05, **P < 0.01 *versus* the compound **6** treatment group

In a previous report, we documented pyrazolo[3,4-*b*] pyridine derivatives that showed potent activity against pulmonary vascular remodeling (Fig. 4A) [14]. To underpin the biological mechanism of these chemicals, we investigated their effects on the interaction between human Hsp110 and ATP. Nevertheless, the screening assay indicated that these compounds, when assayed at the concentration of 100 µM, did not appear to influence the interaction between Hsp110 and ATP (Supporting Information Fig. S1). Given the pathogenic effect of activated STAT3 after forming a complex with Hsp110 in the progression of cancer and the advantage of PPI inhibitors in the treatment of diseases [22, 28, 29], we preliminarily evaluated their activities on the downstream STAT3 pathway in HPAECs. The results revealed that some compounds (compounds below the black dotted line exhibit statistical significance in Fig. 4B, C) could visually reduce the protein levels of p-STAT3 and c-Myc at a concentration of 10 µM, possibly by interfering with the Hsp110/STAT3 interaction (Fig. 4B, C, Supporting Information Fig. S2,3). In addition, considering the good performance of the Hsp110 anti-aggregation assay in high throughput screening [29], we next screened this compound library to explore the candidate that was synchronously active for inhibiting human Hsp110 molecular chaperone function. In this campaign, three structural related compounds (**5**, **6** and **8**) were singled out that reduced the Hsp110 anti-aggregating activity by more than 50% at 100 µM (Fig. 4D). Combining the data from the two selection experiments above, compound **6** showed optimal effects and was therefore selected as the focus of further investigation.

To further evaluate the effect of compound **6** on Hsp110 molecular chaperone function, we tested the degree of luciferase denatures and aggregates under 42 °C at different time points. Addition of Hsp110 drastically reduced the aggregation of luciferase with the aid of its molecular chaperone activity. Correspondingly, the aforehand treatment of Hsp110 with compound **6** significantly increased luciferase degeneration in a time-dependent way, indicating a solid inhibitory effect of **6** on the function of Hsp110 (Fig. 4E). To verify the direct binding between compound **6** and Hsp110, a surface plasmon resonance (SPR) assay was performed on purified recombinant Hsp110. As shown in Fig. 4F, a dose-dependent interaction between Hsp110 and compound **6** was obviously observed in contrast to KNK437 (Supporting Information Fig. S4), an inhibitor of Hsp110 expression [43]. Moreover, drug affinity responsive target stability (DARTS) experimental results further demonstrated that compound **6** could bind to Hsp110 in HPAECs (Fig. 4G, H). In order to determine whether the association could be affected subsequent to **6** treatment, co-immunoprecipitation of endogenous proteins was carried out in HCT116 cells, which have been well documented for Hsp110-STAT3 interaction [29]. The results were shown in Fig. 4I, the association of Hsp110 with STAT3 was strongly decreased after treatment with 10 µM of **6**, whereas compound KNK437 had no effect at 20 µM. Similar results were also obtained when the assay was performed in HPAECs (Fig. 4J), indicating that the interaction of cellular endogenous Hsp110 to STAT3 was significantly reduced after compound **6** directly combined with Hsp110.

### Compound 6 exhibits potent ant-proliferation activity in HPAECs phenotypes via disrupting Hsp110-STAT3 interaction

**Fig. 5:**
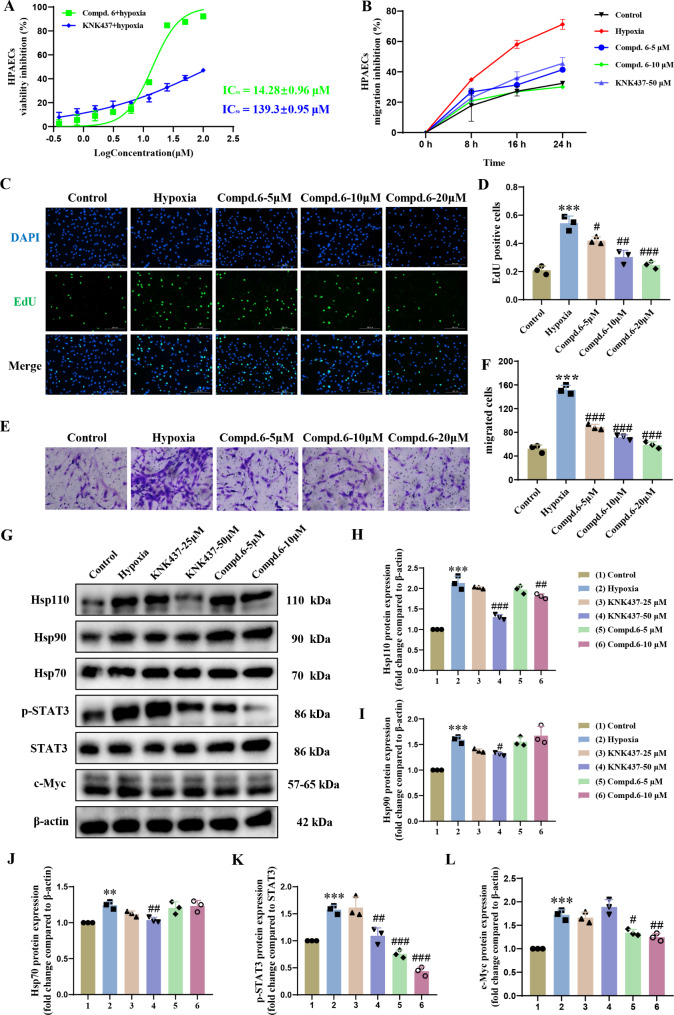
Compound **6** inhibits the abnormal proliferation and migration via disrupting the interaction between Hsp110 and STAT3 under hypoxia. (**A**) Cell viability was assessed by the CCK8 method after treatment with compound **6** or KNK437 under hypoxia for 48 hours. (B) The effect of different concentrations of compound **6** on the migration of HPAECs at different time points were examined by scratch assays. (C) Images of proliferating nuclei labeled with 5-ethynyl-2’-deoxyuridine (EdU) in HPAECs treatment with compound **6** under hypoxia. Scale bars: 200 μm. (D) Statistical analysis of EdU assay. (E) Representative migration images from the trans-well assay in HPAECs treated with compound **6** under hypoxia. Scale bars: 200 μm. (F) Statistical analysis of trans-well assay. (G) Western blots of Hsp110, Hsp90, Hsp70, p-STAT3, STAT3, c-Myc in HPAECs treatment with compound **6** or KNK437 under hypoxia. (H-L): Statistical analysis of western blotting results. Results are expressed as the mean ± standard error, *n* = 3; **P < 0.01, ***P < 0.001 *versus* the control group, ^#^*P* < 0.05, ^##^*P* < 0.01, ^###^*P* < 0.001 *versus* the hypoxia group

As described earlier, elevated Hsp110 has been proved to accelerate abnormal proliferation and migration of HPAECs through favoring STAT3 phosphorylation. To evaluate the efficacy of **6** on adverse cell phenotypes by decreasing Hsp110/STAT3 association, CCK-8 and scratch experiments were performed. As shown in Fig. 5A, compound **6** (IC_50_ = 14.28 ± 0.96 µM) could significantly suppress the over-proliferation of HPAECs in a dose-dependent manner from 0.4 to 100 µM. Furthermore, **6** markedly weakened HPAECs migration in a time- dependent way within 24 h as shown in Fig. 5B. Moreover, the EdU (Fig. 5C-D) and trans-well (Fig. 5E-F) assay results consistently demonstrated strong activity against cell phenotypes, suggesting that **6** is most likely to inhibit the hyperplasia and anomalous migration of HPAECs by attenuating the binding of Hsp110 to STAT3.

In order to further determine the mechanism by which compound **6** regulates the abnormal phenotypes of HPAECs, we carried out the western blotting using KNK437 as the control. The results (Fig. 5G) showed that the protein levels of HSPs, p-STAT3 and c-Myc in HPAECs were significantly increased after hypoxia stimulation. Treatment with compound **6** (10 µM) selectively reduced the protein level of Hsp110 in a mild way (Fig. 5H) without effects on Hsp90 (Fig. 5I) and Hsp70 (Fig. 5G), both of which affect the protein stability and expression of total STAT3 [44], compared with Hsp110 expression inhibitor KNK437 (Fig. 5H-G). Nevertheless, phosphorylated STAT3 and c-Myc levels were significantly lowered after treatment with different concentrations of **6** (5 µM, 10 µM, Fig. 5K, L), which was dramatically stronger than treatment with KNK437. Studies have shown that there is a feedback amplification loop under abnormal conditions where elevated Hsp110 induces STAT3 activation, which in turn aggravates Hsp110 expression via inducing its transcription [22]. The RT-qPCR results showed that compound **6** could reduce the mRNA level of Hsp110 (Supporting Information Fig. S5), indicating that compound **6** might break this positive feedback by reducing the p-STAT3, which caused Hsp110 downregulation through weakening its transcription. In short, the above experimental results fully indicated that compound **6** could significantly inhibit the malignant phenotypes of HPAECs via interfering with the Hsp110-STAT3 interaction.

### Compound 6 effectively mitigates the progression of vascular remodeling in hypoxia-induced PAH rat models

**Fig. 6:**
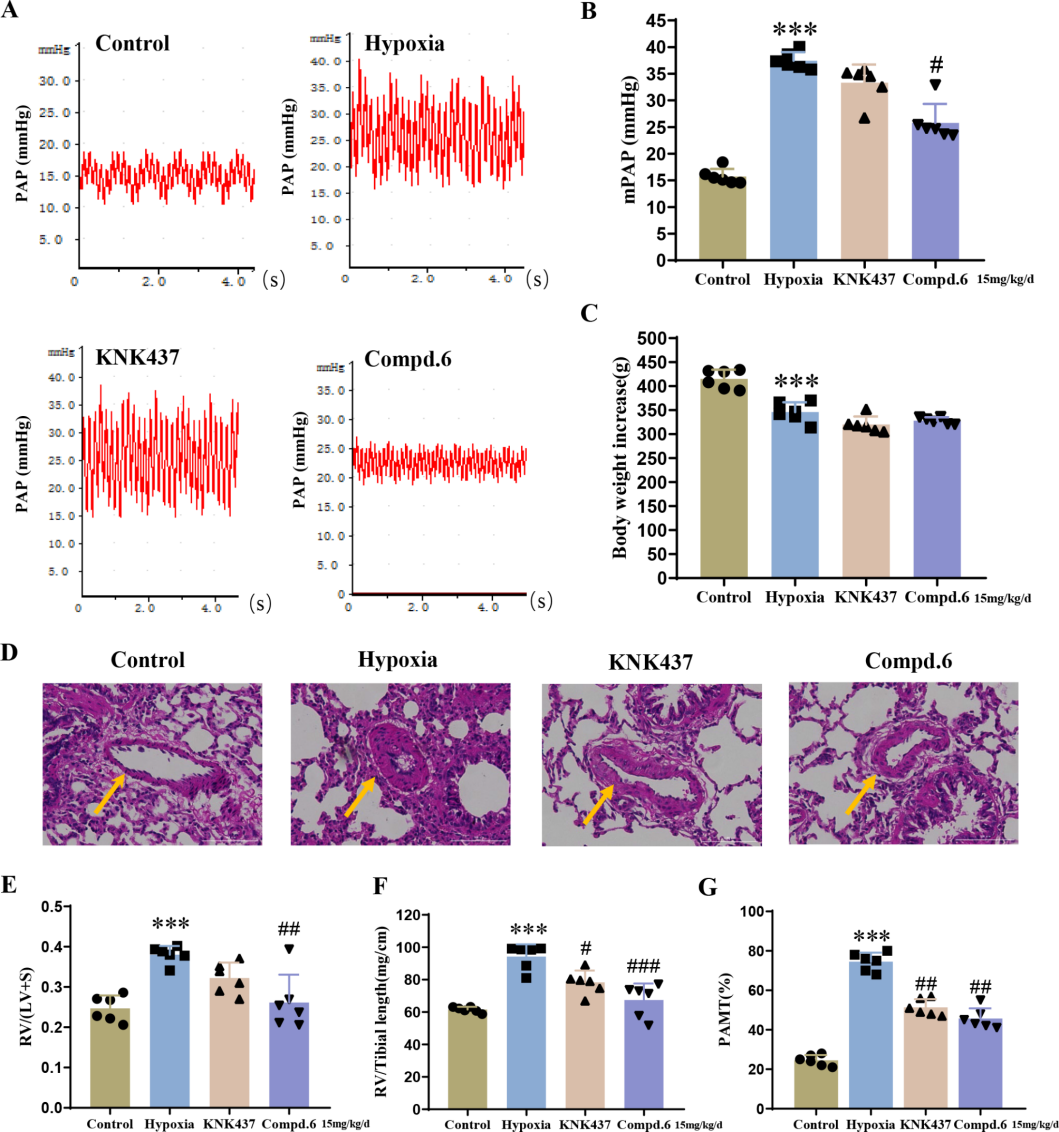
Compound **6** prevents the development of hypoxia-induced PAH. (**A**) mPAP waveform. (**B**) Statistical analysis of mPAP. (**C**) Statistical analysis of body weight (**D**) The representative photographs of hematoxylin and eosin (H&E) staining lung sections from each group. Scale bar: 100 μm. (**E**) RV/(LV + S) statistic. (**F**) RV/tibia length statistic. (**G**) Statistical analysis of PAMT%. Data are expressed as mean ± standard error, *n* = 6. ***P < 0.001 *versus* the control group, ^#^*P* < 0.05, ^##^*P* < 0.01, ^###^*P* < 0.001 *versus* the hypoxia group. mPAP: mean pulmonary artery pressure; RV/(LV + S): right ventricle/(left ventricle + interventricular septum); PAMT: pulmonary artery medial thickness

In view of the good activities of compound **6**in vitro, a hypoxia-induced PAH rat model by hypoxia was established to investigate the pharmacodynamics in vivo. Compound **6** and KNK437 at an equimolar dose (15 mg/kg) were orally administrated daily for 3 weeks under hypoxia (10% O_2_) after the rats had been exposed to continuous hypoxia for 2 weeks. Consistent with the previous results [14], the mPAP (Fig. 6A) of the hypoxia model group was significantly increased compared with the control group, indicating the successful establishment of PAH rat models after 4 weeks of exposure to the hypoxic condition. As shown in Fig. 6B, the administration of **6** remarkably inhibited the elevation of hypoxia-induced mPAP compared to the model group. Meanwhile, treatment with **6** did not cause significant changes in rats body weight, suggesting the low toxic effects for compound **6** (Fig. 6C). In addition, right ventricular hypertrophy (RVH) and pulmonary artery medial thickness (PAMT) were abnormally exacerbated in the PAH model compared to the normoxia group (Fig. 6D-G). In particular, the use of compound **6** and KNK437 appeared to show a pronounced effect on delaying the process of pulmonary vascular remodeling and RVH induced by hypoxia. Taken together, these results indicate that compound **6** may inactivate the STAT3 signaling pathway through blocking the binding of Hsp110 to STAT3, thus inhibiting the pathological changes of pulmonary vascular remodeling in vivo.

### Compound 6 effectively inhibits Hsp110-STAT3 interaction in hypoxia-induced PAH rats

**Fig. 7:**
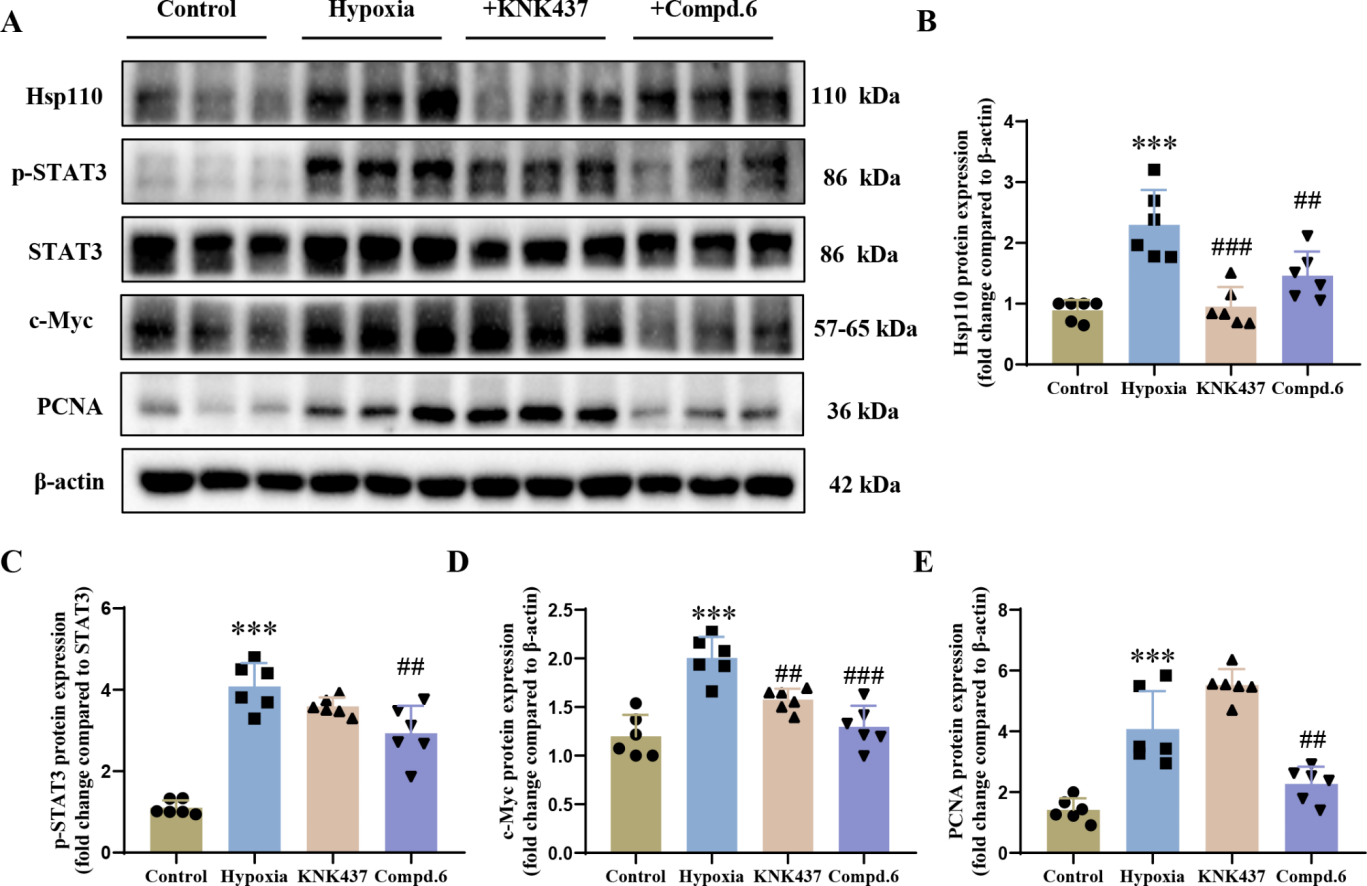
Compound **6** inhibited Hsp110-STAT3 PPI in hypoxia-induced PAH rats. (**A**): Western blots of Hsp110, p-STAT3, STAT3, c-Myc, PCNA in pulmonary arteries of PAH rats after compound **6** or KNK437 treatment. (**B**) Statistical analysis of Hsp110 expression in rat pulmonary arteries. (**C**) Statistical analysis of p-STAT3/STAT3 ratio in rat pulmonary arteries. (**D**) Statistical analysis of c-Myc levels in rat pulmonary arteries. (**E**) Statistical analysis of PCNA levels in rat pulmonary arteries. Data are expressed as mean ± standard error, *n* = 6. ***P < 0.001 *versus* the control group, ^##^*P* < 0.01, ^###^*P* < 0.001 *versus* the hypoxia group

To further confirm the mechanism of **6** in improving vascular remodeling in vivo, we measured the levels of related downstream proteins of Hsp110-STAT3 PPI activation. Specifically, we mainly assessed changes in the protein levels of p-STAT3 and c-Myc after administration in the pulmonary artery of hypoxia-induced PAH rat models. As shown in Fig. 7A, compound **6** and KNK437 observably attenuated Hsp110 protein expression stimulated by hypoxia (Fig. 7B), which is in accordance with the influences in HPAECs. Strikingly, treatment with **6** more significantly decreased the levels of p-STAT3 and c-Myc without obviously changing the total levels of STAT3 in PAH rat pulmonary arteries induced under hypoxia compared to the KNK437 treatment group (Fig. 7C, D). In addition, compound **6** markedly reduced the protein level of proliferating cell nuclear antigen (PCNA), a representative marker of vascular remodeling, while KNK437 showed no obvious effect (Fig. 7E). In a word, these results revealed that compound **6** could reduce the downstream protein levels of p-STAT3 and c-Myc by interfering with the interaction between Hsp110 and STAT3, thereby ameliorating pulmonary vascular remodeling in hypoxia-induced PAH rat models.

**Fig. 8:**
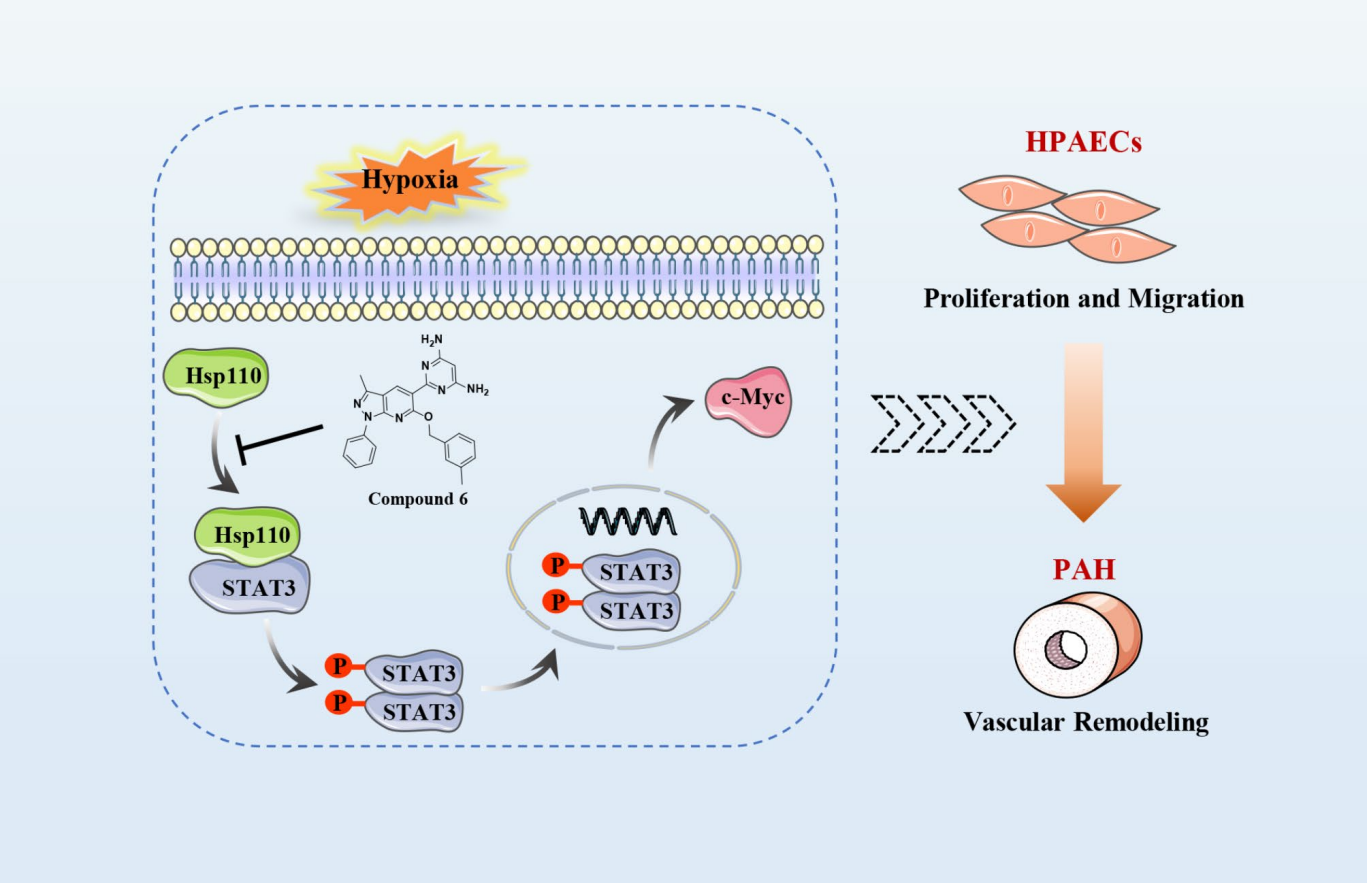
The molecular mechanism of Hsp110 in endothelial dysfunction and the mode of action of compound **6** in treating PAH. The abnormal up-regulation of Hsp110 induced by hypoxia plays an important role in the pathogenesis of pulmonary vascular remodeling and PAH development. In HPAECs, compound **6** interferes with the interaction between Hsp110 and STAT3 through direct binding to Hsp110, decreasing the protein levels of p-STAT3 and c-Myc, thereby inhibiting the abnormal proliferation and migration of endothelial cells and affecting endothelial function, ultimately improving pulmonary vascular remodeling

## Discussion

In the present study, we investigated for the first time the role of Hsp110 in pulmonary vascular remodeling, which led us to the following key observations: (1) Hsp110 levels are elevated in the serum of PAH patients and in the lungs and pulmonary arteries of hypoxia-induced PAH rats. (2) Hsp110 was mainly localized in CD31-positive ECs and was significantly increased under hypoxia stimulation. (3) boosted Hsp110-STAT3 interaction under hypoxia induced HPAECs abnormal proliferation and migration via elevating p-STAT3 and c-Myc levels. (4) we successfully screened Hsp110-STAT3 PPI inhibitor compound **6** which effectively inhibited HPAECs proliferation and migration, improved vascular remodeling of PAH rats. Our studies preliminarily confirmed that Hsp110 plays an important role in the early stage of PAH development and could be used as a novel potential target for improving vascular remodeling.

The currently approved first-line drugs for PAH treatment including prostacyclin analogs, endothelin receptor antagonists and NO pathways regulators, have greatly improved the quality of PAH patient’s life through dilating pulmonary blood vessels [45]. However, these vasodilators are running into drawbacks and bottlenecks, such as systematic hypotension and drug resistance [15]. During PAH development, the physiological changes enable pulmonary vascular cells to acquire a cancer-like phenotype including hyperproliferation and resistance to apoptosis, thus resulting in pulmonary vascular remodeling that was regarded as the essential pathological characteristic of PAH [46]. Therefore, vascular remodeling is attracting more and more attention, and offers an opportunity to exploit new therapeutic targets in the field of drug discovery for PAH [47].

Nowadays, novel drugs in preclinical and clinical research mainly focus on pulmonary vascular remodeling such as G-protein-coupled receptors, growth factor receptors, transcription factors, and HSPs, as we reviewed earlier [15]. At present, the aromatase inhibitor Anastrozole, estrogen receptor α inhibitor Fulvestrant and novel nitro fatty acid CXA-10 have all entered the phase 2 clinical study against PAH [48–50]. Remarkable, as a selective TGF-β ligand trap, Sotatercept significantly improved vascular remodeling induced by hypoxia and MCT. Currently, Merck has completed the Phase 3 clinical trial for Sotatercept and submitted a Biologics License Application to the U.S. Food and Drug Administration (FDA) for the treatment of PAH [51]. Recently, numerous studies have confirmed that endothelial dysfunction, such as hyperplasia and abnormal migration of HPAECs, is the initial trigger in pulmonary vascular remodeling [5]. Recent studies have well shown that targeting the early stage of pulmonary vascular remodeling is a promising direction for curing PAH [6–9]. However, current therapeutic targets and drugs under study for improving endothelial dysfunction are extremely limited. Thus, it holds great promising to explore new therapeutic strategy targeting the early stage of pulmonary vascular remodeling for PAH treatment.

Heat shock proteins are a class of proteins that are dysregulated in the process of vascular remodeling, and inhibition of HSPs is emerging as a promising strategy for PAH targeted therapy [52]. Notably, the latest recent research showed that high expression of Hsp110 is involved in the regulation of uncontrolled proliferation and migration of HPASMCs [24]. In this study, we found that Hsp110 is more highly expressed in HPAECs compared with HPASMCs. Nevertheless, as a stress protein, the role and regulatory mechanism of Hsp110 in endothelial dysfunction and further vascular remodeling remains to be elucidated. In order to preliminarily investigate the role of Hsp110 in the early stage of PAH development, we conducted relevant studies in HPAECs. Intriguingly, our results revealed that Hsp110 plays an important role in regulating endothelial dysfunction and thus inducing vascular remodeling. Inspired by the pathogenic mechanism of Hsp110 in oncology, we confirmed for the first time that enhanced interaction between Hsp110 and STAT3 leads to abnormal phenotypes of HPAECs via activation of downstream STAT3 signaling pathway. Moreover, specific functional modulation by PPI inhibitors could achieve therapeutic effects without the side effects of heat shock response and high toxicity caused by directly inhibiting the ATPase activity of HSPs [27]. These results suggest the potential effect of Hsp110-STAT3 PPI inhibition as a novel strategy to improve vascular remodeling. Apparently, Hsp110/STAT3 PPI inhibitors represent the first-in-class drug against PAH. However, no small molecule has been reported to play a therapeutic role for PAH by regulating Hsp110/STAT3 PPI up to now.

Based on the existing research results, we screened the library of pyrazolo[3,4-*b*] pyridine derivatives that possess anti-vascular remodeling activities [14]. We comprehensively evaluated their activity on Hsp110 anti-aggregation effect and activated STAT3 pathway, which finally directed us to focus on compound **6**. Through many in vitro assays such as SPR, DARTS and Co-IP, we have fully demonstrated that compound **6** could manage the abnormal phenotypes of HPAECs by interfering with the Hsp110-STAT3 PPI and then reducing the protein levels of p-STAT3 and c-Myc. Moreover, pharmacodynamic studies in vivo showed that compound **6** could significantly inhibit pulmonary vascular remodeling, improve RVH, and reduce the mPAP in hypoxia-induced PAH rats via disrupting the Hsp110-STAT3 interaction. Taken together, our results in this work preliminarily proved that compound **6** could be a new lead compound for drug discovery against PAH by targeting Hsp110-STAT3 interaction.

## Conclusions

Our study first validated that Hsp110 was up-regulated in human serum and rat’s tissues with PAH, and overexpression of Hsp110 promoted HPAECs proliferation and migration. Furthermore, knockdown of Hsp110 significantly inhibited the abnormal phenotypes of HPAECs through suppressing the downstream STAT3 signaling pathway. Notably, we successfully identified compound **6** as a promising lead compound that disrupts the Hsp110-STAT3 interaction, resulting in the reduction of p-STAT3 and c-Myc levels. Moreover, compound **6** improved endothelial dysfunction and blocked vascular remodeling in our in vitro and in vivo PAH models. Our results suggest that disruption of the Hsp110-STAT3 association exhibits therapeutic effects against PAH progression, and provide a promising lead compound to develop the first-in-class agent targeting Hsp110-STAT3 PPI for PAH treatment.

### Electronic supplementary material

Below is the link to the electronic supplementary material.

Supplementary Material 1

## Data Availability

Not applicable.

## Abbreviations

HPAECs: Human pulmonary arterial endothelial cells
HPASMCs: Human pulmonary arterial smooth muscle cells
Hsp110: Heat shock protein 110
HSPs: Heat shock proteins
MCT: Monocrotaline
PAH: Pulmonary arterial hypertension
PAP: Pulmonary arterial pressure
PCNA: Proliferating cell nuclear antigen
PPI: Protein-protein interaction
RVH: Right ventricular hypertrophy
PVR: Pulmonary vascular resistance
STAT3: Signal transducer and activator of transcription 3
