# Supplementary material for: Inhibition of Hsp110-STAT3 interaction in endothelial cells alleviates vascular remodeling in hypoxic pulmonary arterial Hypertension model
Source: Respir Res. 2023 Nov 17;24:289. doi: 10.1186/s12931-023-02600-5 (PMC10655391; doi:10.1186/s12931-023-02600-5)
Supplement: Supplementary file 1 — Supplementary Material 1 [file 12931_2023_2600_MOESM1_ESM.pdf]

Inhibition of Hsp110-STAT3 interaction in endothelial cells alleviates vascular remodeling in hypoxic pulmonary arterial hypertension model

Congke Zhao[a,c,d,1], Xiangyang Le[a,c,d,1], Mengqi Li[a,c,d], Yuanbo Hu[a,c,d], Xiaohui Li[e], Zhuo Chen[a,c,d], Gaoyun Hu[a,c,d], Liqing Hu[b*] and Qianbin Li[a,c,d,*]

[a] *Department of Medicinal Chemistry, Xiangya School of Pharmaceutical Sciences, Central South University, Changsha 410013, Hunan, China*
[b] *Key Laboratory of Study and Discovery of Small Targeted Molecules of Hunan Province, Department of Pharmacy, School of Medicine, Hunan Normal University, Changsha 410013, Hunan, China*
[c] *Hunan Key Laboratory of Diagnostic and Therapeutic Drug Research for Chronic Diseases, Changsha 410013, Hunan, China*
[d] *Hunan Key Laboratory of Organ Fibrosis, Changsha 410013, Hunan, China*
[e] *Department of Pharmacology, Xiangya School of Pharmaceutical Sciences, Central South University, Changsha 410013, Hunan, China*

Corresponding author: ***Qianbin Li****, qbli@csu.edu.cn;* ***Liqing Hu****, huliqing@hunnu.edu.cn*

[1]These authors made equal contributions to this work.

Table of contents:

Figure S1 Compound screening results for the human Hsp110 and ATP binding activity at 100 μM……………………………………………………………………….…………S2

FigureS2 Compound structures and screening results targeting Hsp110/STAT3 signaling at 10 μM……………………………………………………………………….……………………S2

Figure S3 Compound screening results targeting the expression of Hsp110 at 10 μM……………………………………………………………………………………..S3

Figure S4 SPR assay for the interaction between KNK437and Hsp110……………S3

Figure S5 The effects of compound **6** on the mRNA levels of Hsp110 in PAECs………..S3

Table S1 Primers used for real-time RT-qPCR………………………………………..S4

Original western blots………………………………………………………………S5-S9

Figure S1

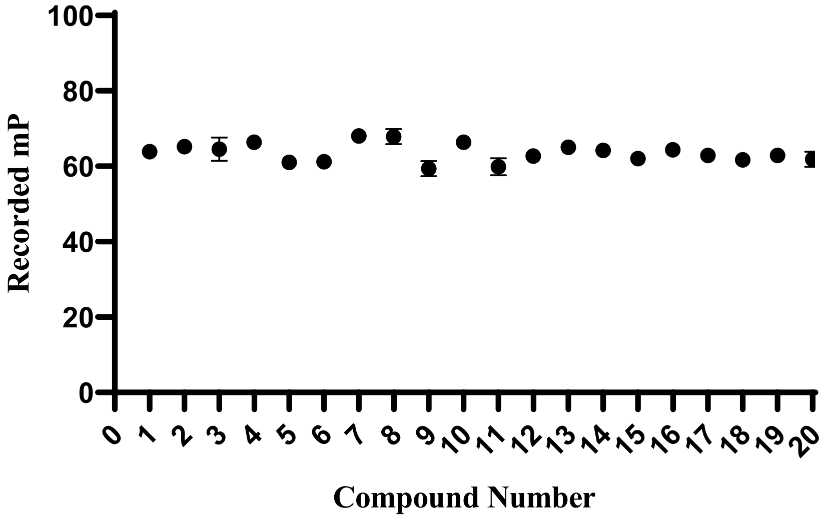

Compound screening results for the human Hsp110 and ATP binding activity at 100 μM.

Figure S2

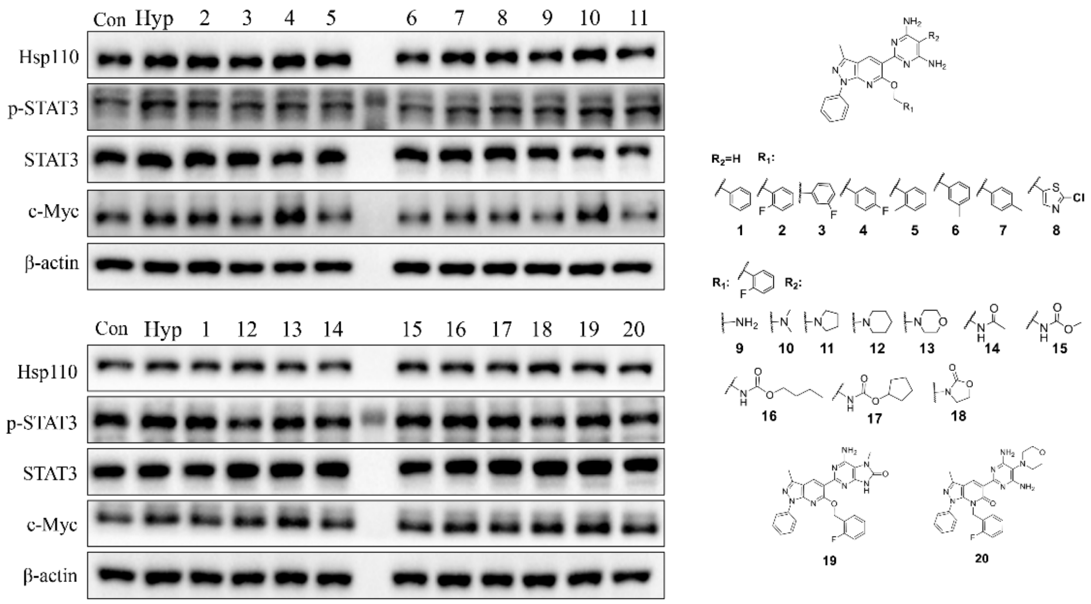

Compound structures and screening results targeting Hsp110/STAT3 signaling at 10 μM. Western blot analysis of Hsp110, p-STAT3, STAT3 and c-Myc expression level in HPAECs. Results are expressed as the mean ± standard error, $n = 3$; $^{**}P < 0.05$ *versus* the hypoxia group.

Figure S3

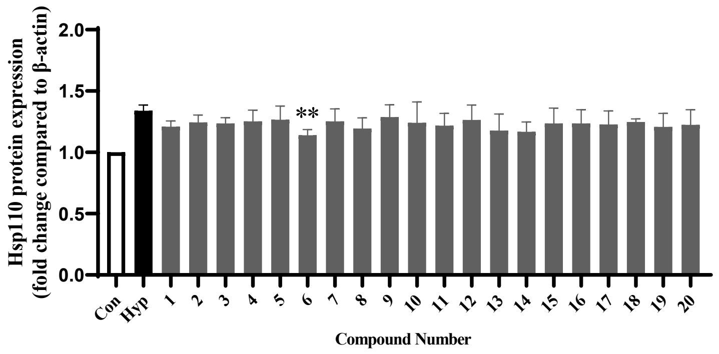

Compound screening results targeting the expression of Hsp110 at 10 μM. Results are expressed as the mean ± standard error, $n = 3$; $^{**}P < 0.05$ *versus* the hypoxia group.

Figure S4

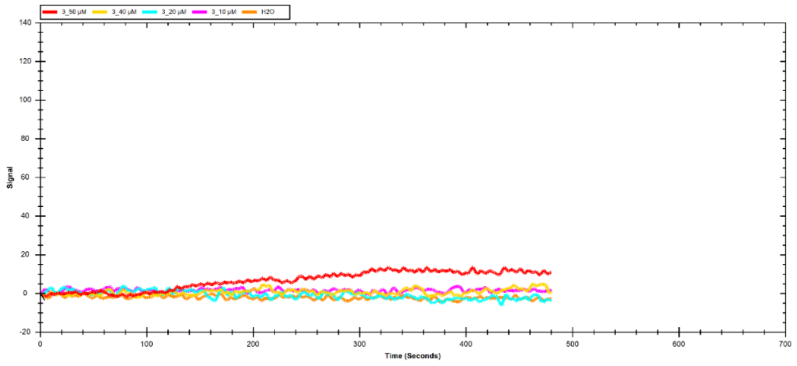

SPR assay for the interaction between KNK437and Hsp110.

Figure S5

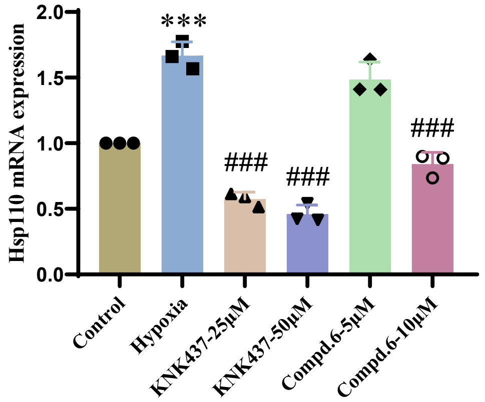

The effects of compound 6 on the mRNA levels of Hsp110 in PAECs. Results are expressed as the mean ± standard error, $n = 3$; $^{***}P < 0.001$ *versus* the control group, $^{\#\#\#}P < 0.001$ *versus* the hypoxia group.

Table S1. Primers used for real-time RT-qPCR

Gene	Forward primer	Reverse primer
Hsp110	5′-ATGAAGTGATGGAATGGATG-3′	5′-TTTGGTTTCGGTTGTGTTAC-3′
GAPDH	5′- AACGGATTTGGTCGTATTGGG-3′	5′- CCTGGAAGATGGTGATGGGAT-3′

Original western blots

Fig. 1

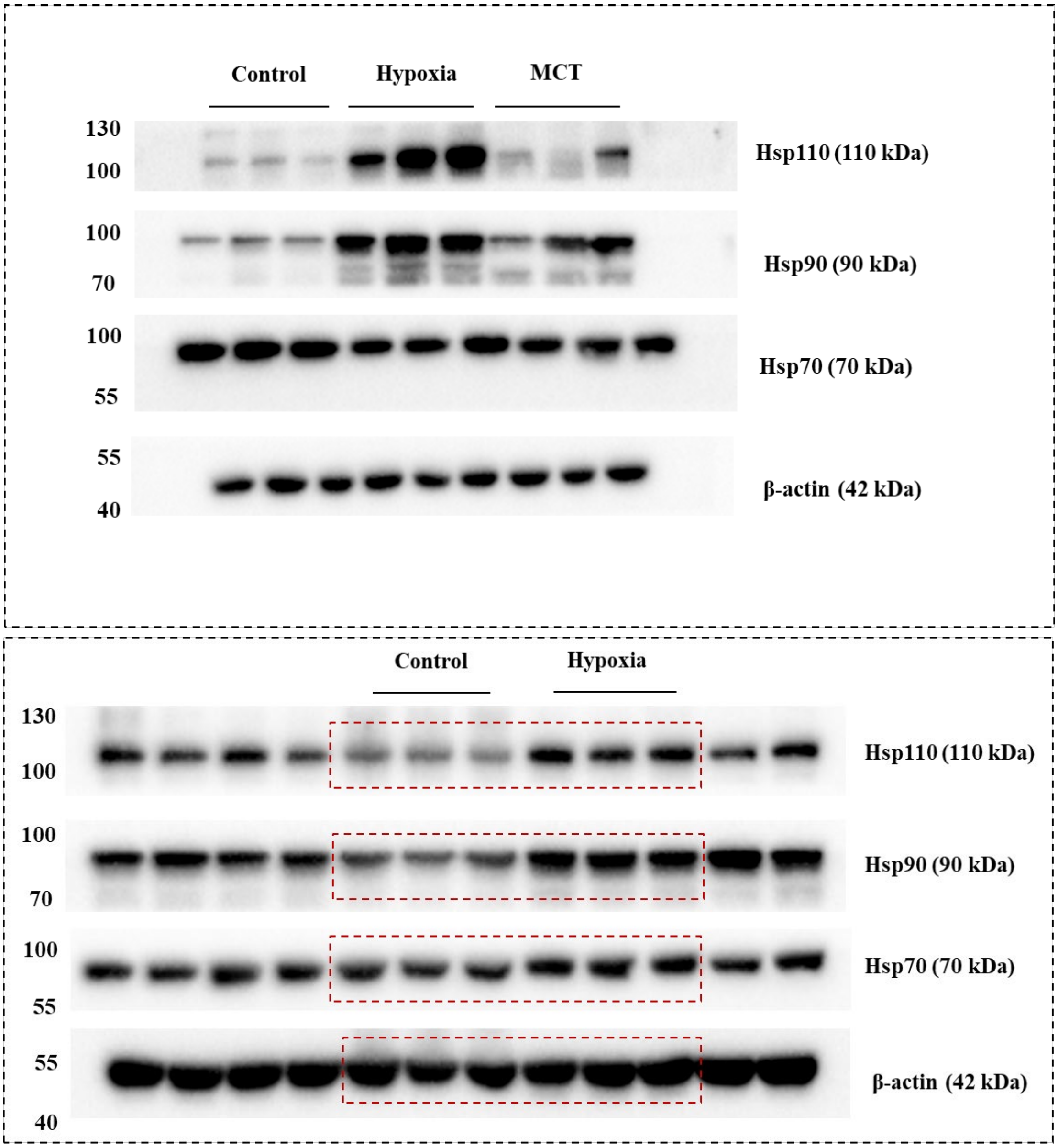

Fig. 2

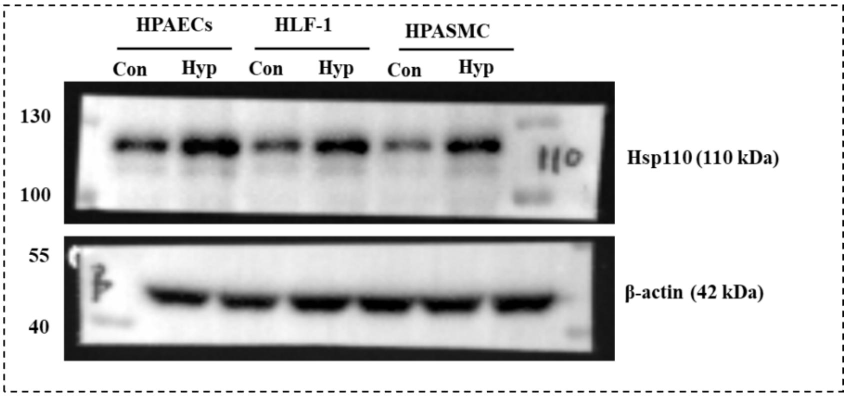
HPAECs
HLF-1
HPASMC
Con
Hyp
Con
Hyp
Con
Hyp
130
100
Hsp110 (110 kDa)
55
40
β-actin (42 kDa)

Fig. 3

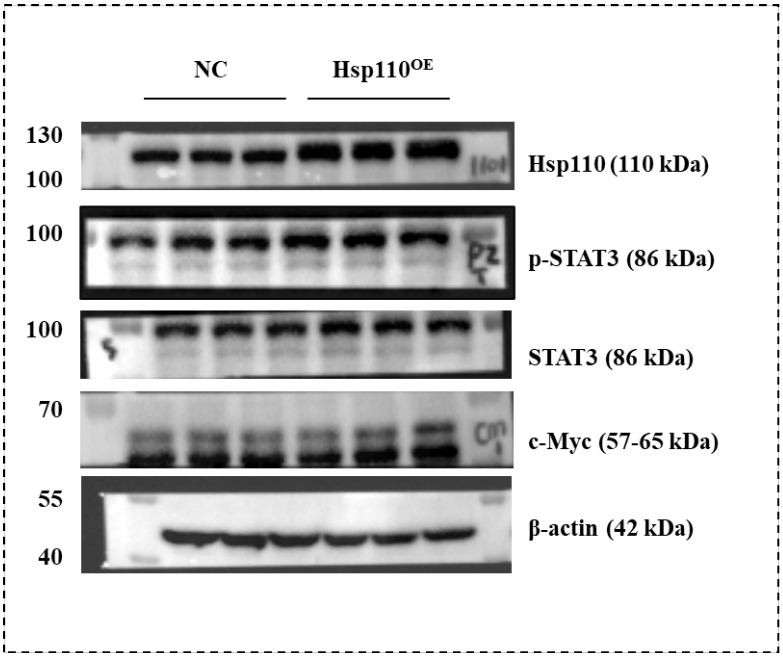
NC
Hsp110OE
130
100
Hsp110 (110 kDa)
100
p-STAT3 (86 kDa)
100
STAT3 (86 kDa)
70
c-Myc (57-65 kDa)
55
40
β-actin (42 kDa)

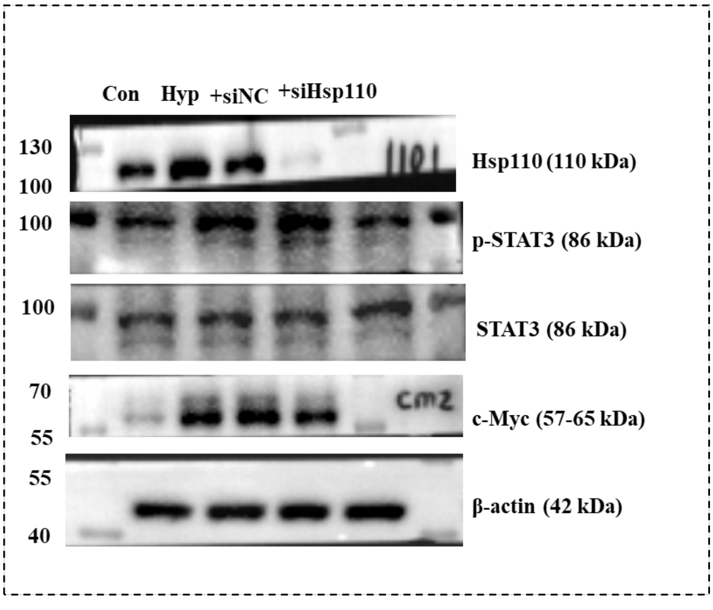
Con
Hyp
+siNC
+siHsp110
130
100
Hsp110 (110 kDa)
100
p-STAT3 (86 kDa)
100
STAT3 (86 kDa)
70
55
c-Myc (57-65 kDa)
55
40
β-actin (42 kDa)

Fig. 4

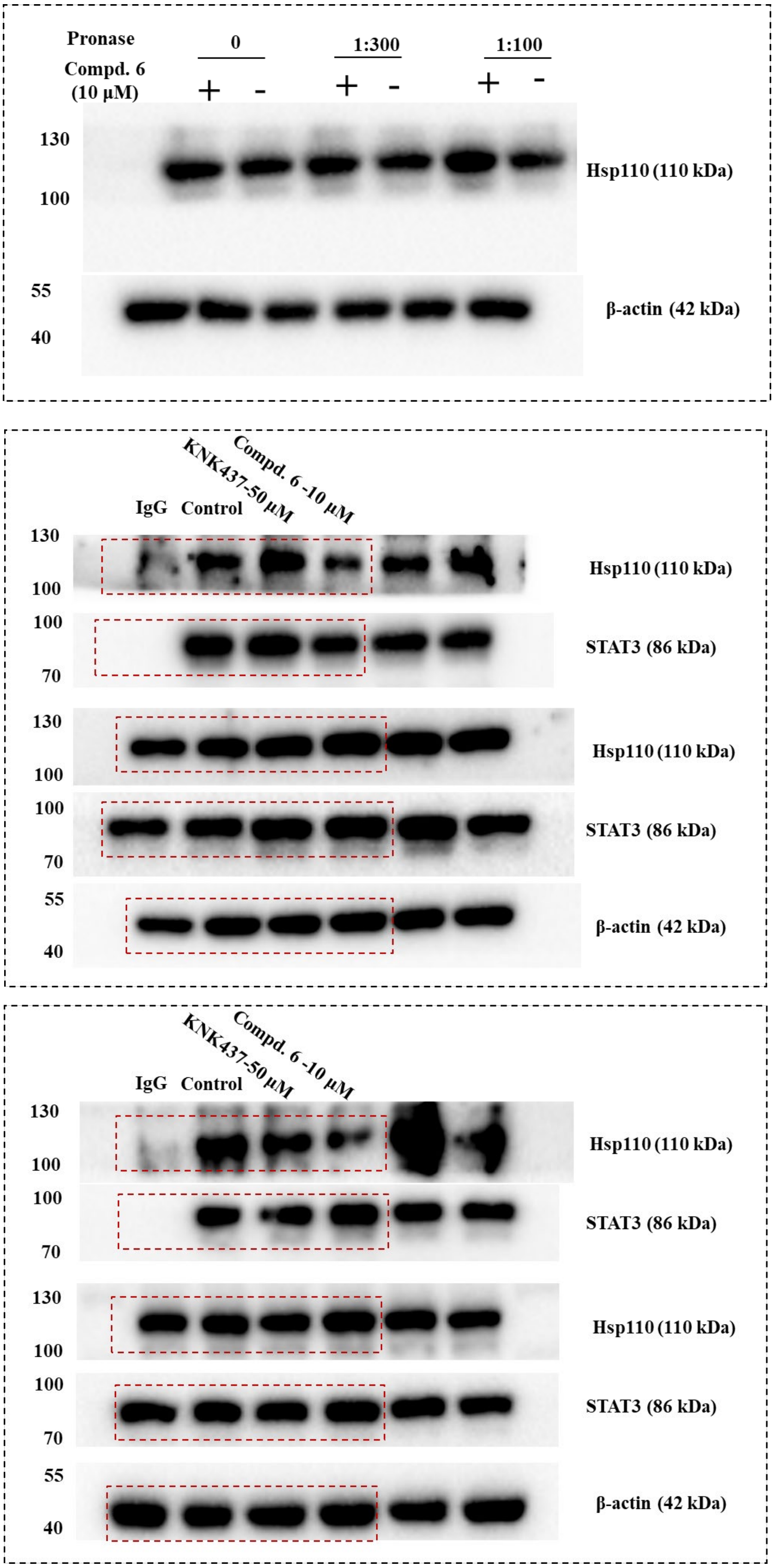

Pronase
0
1:300
1:100
Compd. 6
(10 μM)
+
-
+
-
+
-
130
100
Hsp110 (110 kDa)
55
40
β-actin (42 kDa)
IgG
Control
KNK437-50 μM
Compd. 6 -10 μM
130
100
Hsp110 (110 kDa)
100
70
STAT3 (86 kDa)
130
100
Hsp110 (110 kDa)
100
70
STAT3 (86 kDa)
55
40
β-actin (42 kDa)
IgG
Control
KNK437-50 μM
Compd. 6 -10 μM
130
100
Hsp110 (110 kDa)
100
70
STAT3 (86 kDa)
130
100
Hsp110 (110 kDa)
100
70
STAT3 (86 kDa)
55
40
β-actin (42 kDa)

Fig. 5

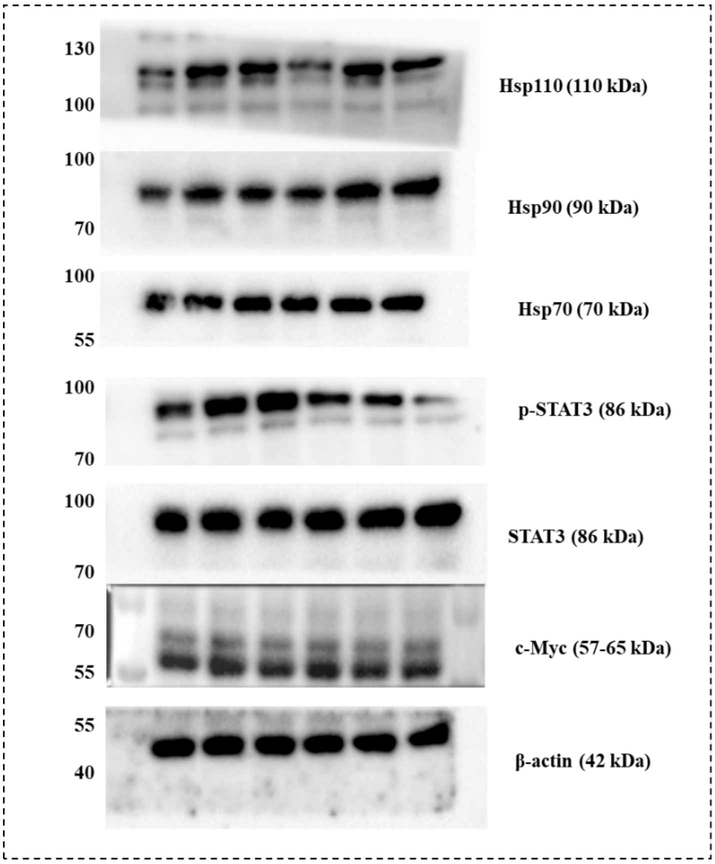
130
100
Hsp110 (110 kDa)
100
70
Hsp90 (90 kDa)
100
55
Hsp70 (70 kDa)
100
70
p-STAT3 (86 kDa)
100
70
STAT3 (86 kDa)
70
55
c-Myc (57-65 kDa)
55
40
β-actin (42 kDa)

Fig. 7

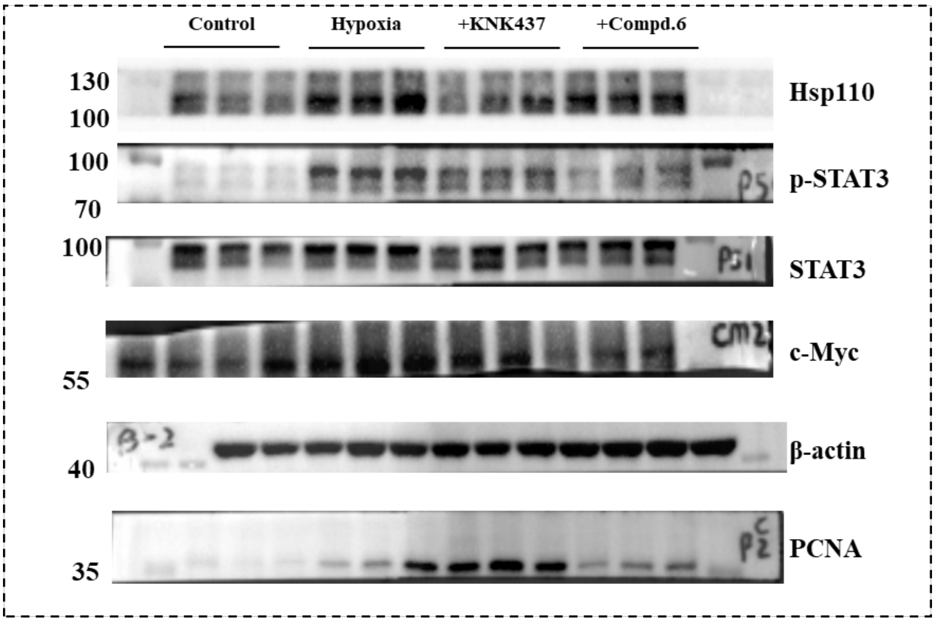
Control
Hypoxia
+KNK437
+Compd.6
130
100
Hsp110
100
70
p-STAT3
100
STAT3
55
c-Myc
40
β-actin
35
PCNA

Figure S2.

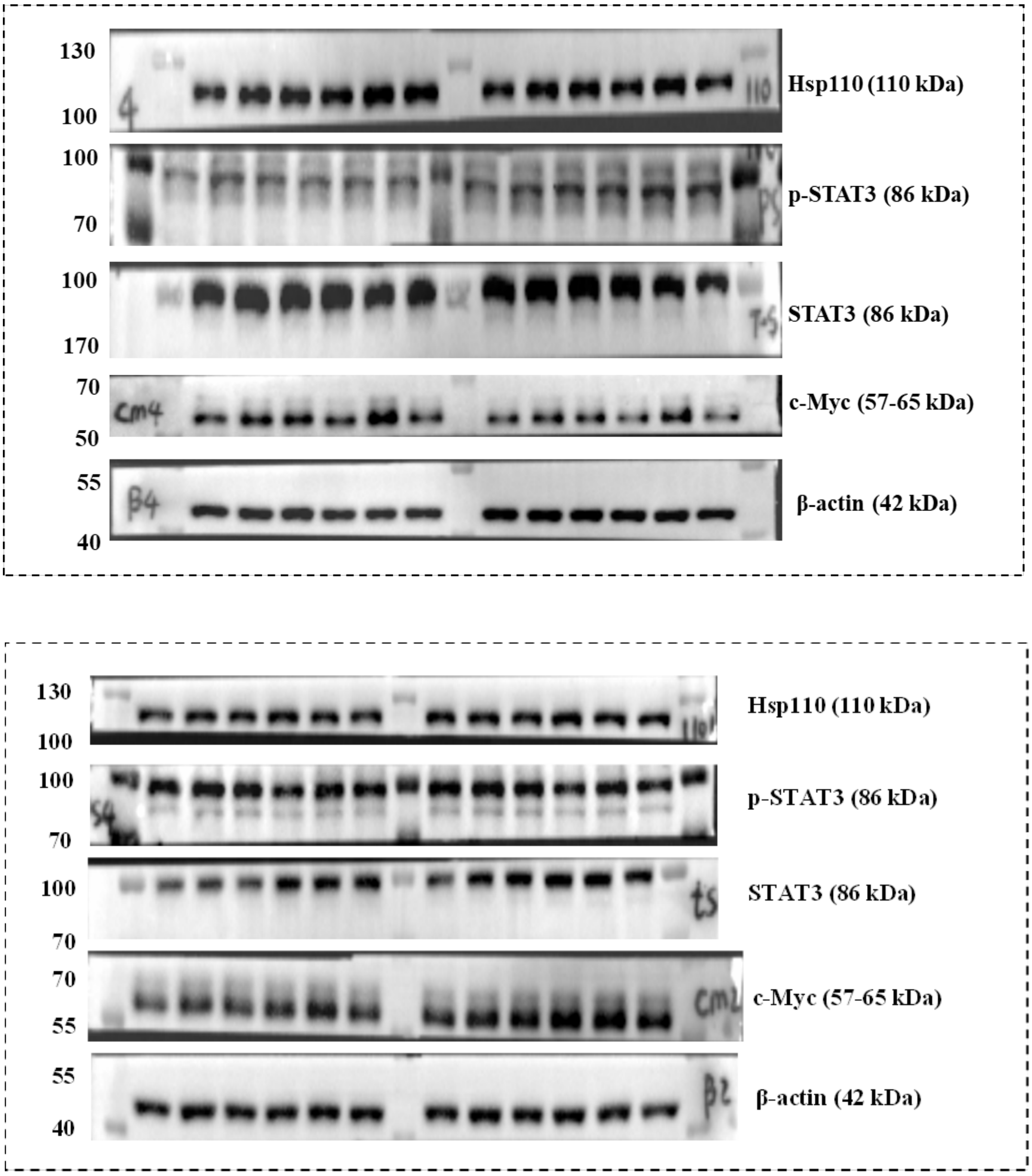
130
100
Hsp110 (110 kDa)
100
70
p-STAT3 (86 kDa)
100
170
STAT3 (86 kDa)
70
50
c-Myc (57-65 kDa)
55
40
β-actin (42 kDa)
130
100
Hsp110 (110 kDa)
100
70
p-STAT3 (86 kDa)
100
70
STAT3 (86 kDa)
70
55
c-Myc (57-65 kDa)
55
40
β-actin (42 kDa)
